# Combined burden and functional impact tests for cancer driver discovery using DriverPower

**DOI:** 10.1038/s41467-019-13929-1

**Published:** 2020-02-05

**Authors:** Shimin Shuai, PCAWG Drivers and Functional Interpretation Working Group, Federico Abascal, Samirkumar B. Amin, Gary D. Bader, Pratiti Bandopadhayay, Jonathan Barenboim, Rameen Beroukhim, Johanna Bertl, Keith A. Boroevich, Søren Brunak, Peter J. Campbell, Joana Carlevaro-Fita, Dimple Chakravarty, Calvin Wing Yiu Chan, Ken Chen, Jung Kyoon Choi, Jordi Deu-Pons, Priyanka Dhingra, Klev Diamanti, Lars Feuerbach, J. Lynn Fink, Nuno A. Fonseca, Joan Frigola, Carlo Gambacorti-Passerini, Dale W. Garsed, Mark Gerstein, Gad Getz, Qianyun Guo, Ivo G. Gut, David Haan, Mark P. Hamilton, Nicholas J. Haradhvala, Arif O. Harmanci, Mohamed Helmy, Carl Herrmann, Julian M. Hess, Asger Hobolth, Ermin Hodzic, Chen Hong, Henrik Hornshøj, Keren Isaev, Jose M. G. Izarzugaza, Rory Johnson, Todd A. Johnson, Malene Juul, Randi Istrup Juul, Andre Kahles, Abdullah Kahraman, Manolis Kellis, Ekta Khurana, Jaegil Kim, Jong K. Kim, Youngwook Kim, Jan Komorowski, Jan O. Korbel, Sushant Kumar, Andrés Lanzós, Erik Larsson, Michael S. Lawrence, Donghoon Lee, Kjong-Van Lehmann, Shantao Li, Xiaotong Li, Ziao Lin, Eric Minwei Liu, Lucas Lochovsky, Shaoke Lou, Tobias Madsen, Kathleen Marchal, Iñigo Martincorena, Alexander Martinez-Fundichely, Yosef E. Maruvka, Patrick D. McGillivray, William Meyerson, Ferran Muiños, Loris Mularoni, Hidewaki Nakagawa, Morten Muhlig Nielsen, Marta Paczkowska, Keunchil Park, Kiejung Park, Jakob Skou Pedersen, Tirso Pons, Sergio Pulido-Tamayo, Benjamin J. Raphael, Jüri Reimand, Iker Reyes-Salazar, Matthew A. Reyna, Esther Rheinbay, Mark A. Rubin, Carlota Rubio-Perez, S. Cenk Sahinalp, Gordon Saksena, Leonidas Salichos, Chris Sander, Steven E. Schumacher, Mark Shackleton, Ofer Shapira, Ciyue Shen, Raunak Shrestha, Shimin Shuai, Nikos Sidiropoulos, Lina Sieverling, Nasa Sinnott-Armstrong, Lincoln D. Stein, Joshua M. Stuart, David Tamborero, Grace Tiao, Tatsuhiko Tsunoda, Husen M. Umer, Liis Uusküla-Reimand, Alfonso Valencia, Miguel Vazquez, Lieven P. C. Verbeke, Claes Wadelius, Lina Wadi, Jiayin Wang, Jonathan Warrell, Sebastian M. Waszak, Joachim Weischenfeldt, David A. Wheeler, Guanming Wu, Jun Yu, Jing Zhang, Xuanping Zhang, Yan Zhang, Zhongming Zhao, Lihua Zou, Christian von Mering, Steven Gallinger, Lincoln D. Stein, PCAWG Consortium, Lauri A. Aaltonen, Federico Abascal, Adam Abeshouse, Hiroyuki Aburatani, David J. Adams, Nishant Agrawal, Keun Soo Ahn, Sung-Min Ahn, Hiroshi Aikata, Rehan Akbani, Kadir C. Akdemir, Hikmat Al-Ahmadie, Sultan T. Al-Sedairy, Fatima Al-Shahrour, Malik Alawi, Monique Albert, Kenneth Aldape, Ludmil B. Alexandrov, Adrian Ally, Kathryn Alsop, Eva G. Alvarez, Fernanda Amary, Samirkumar B. Amin, Brice Aminou, Ole Ammerpohl, Matthew J. Anderson, Yeng Ang, Davide Antonello, Pavana Anur, Samuel Aparicio, Elizabeth L. Appelbaum, Yasuhito Arai, Axel Aretz, Koji Arihiro, Shun-ichi Ariizumi, Joshua Armenia, Laurent Arnould, Sylvia Asa, Yassen Assenov, Gurnit Atwal, Sietse Aukema, J. Todd Auman, Miriam R. R. Aure, Philip Awadalla, Marta Aymerich, Gary D. Bader, Adrian Baez-Ortega, Matthew H. Bailey, Peter J. Bailey, Miruna Balasundaram, Saianand Balu, Pratiti Bandopadhayay, Rosamonde E. Banks, Stefano Barbi, Andrew P. Barbour, Jonathan Barenboim, Jill Barnholtz-Sloan, Hugh Barr, Elisabet Barrera, John Bartlett, Javier Bartolome, Claudio Bassi, Oliver F. Bathe, Daniel Baumhoer, Prashant Bavi, Stephen B. Baylin, Wojciech Bazant, Duncan Beardsmore, Timothy A. Beck, Sam Behjati, Andreas Behren, Beifang Niu, Cindy Bell, Sergi Beltran, Christopher Benz, Andrew Berchuck, Anke K. Bergmann, Erik N. Bergstrom, Benjamin P. Berman, Daniel M. Berney, Stephan H. Bernhart, Rameen Beroukhim, Mario Berrios, Samantha Bersani, Johanna Bertl, Miguel Betancourt, Vinayak Bhandari, Shriram G. Bhosle, Andrew V. Biankin, Matthias Bieg, Darell Bigner, Hans Binder, Ewan Birney, Michael Birrer, Nidhan K. Biswas, Bodil Bjerkehagen, Tom Bodenheimer, Lori Boice, Giada Bonizzato, Johann S. De Bono, Arnoud Boot, Moiz S. Bootwalla, Ake Borg, Arndt Borkhardt, Keith A. Boroevich, Ivan Borozan, Christoph Borst, Marcus Bosenberg, Mattia Bosio, Jacqueline Boultwood, Guillaume Bourque, Paul C. Boutros, G. Steven Bova, David T. Bowen, Reanne Bowlby, David D. L. Bowtell, Sandrine Boyault, Rich Boyce, Jeffrey Boyd, Alvis Brazma, Paul Brennan, Daniel S. Brewer, Arie B. Brinkman, Robert G. Bristow, Russell R. Broaddus, Jane E. Brock, Malcolm Brock, Annegien Broeks, Angela N. Brooks, Denise Brooks, Benedikt Brors, Søren Brunak, Timothy J. C. Bruxner, Alicia L. Bruzos, Alex Buchanan, Ivo Buchhalter, Christiane Buchholz, Susan Bullman, Hazel Burke, Birgit Burkhardt, Kathleen H. Burns, John Busanovich, Carlos D. Bustamante, Adam P. Butler, Atul J. Butte, Niall J. Byrne, Anne-Lise Børresen-Dale, Samantha J. Caesar-Johnson, Andy Cafferkey, Declan Cahill, Claudia Calabrese, Carlos Caldas, Fabien Calvo, Niedzica Camacho, Peter J. Campbell, Elias Campo, Cinzia Cantù, Shaolong Cao, Thomas E. Carey, Joana Carlevaro-Fita, Rebecca Carlsen, Ivana Cataldo, Mario Cazzola, Jonathan Cebon, Robert Cerfolio, Dianne E. Chadwick, Dimple Chakravarty, Don Chalmers, Calvin Wing Yiu Chan, Kin Chan, Michelle Chan-Seng-Yue, Vishal S. Chandan, David K. Chang, Stephen J. Chanock, Lorraine A. Chantrill, Aurélien Chateigner, Nilanjan Chatterjee, Kazuaki Chayama, Hsiao-Wei Chen, Jieming Chen, Ken Chen, Yiwen Chen, Zhaohong Chen, Andrew D. Cherniack, Jeremy Chien, Yoke-Eng Chiew, Suet-Feung Chin, Juok Cho, Sunghoon Cho, Jung Kyoon Choi, Wan Choi, Christine Chomienne, Zechen Chong, Su Pin Choo, Angela Chou, Angelika N. Christ, Elizabeth L. Christie, Eric Chuah, Carrie Cibulskis, Kristian Cibulskis, Sara Cingarlini, Peter Clapham, Alexander Claviez, Sean Cleary, Nicole Cloonan, Marek Cmero, Colin C. Collins, Ashton A. Connor, Susanna L. Cooke, Colin S. Cooper, Leslie Cope, Vincenzo Corbo, Matthew G. Cordes, Stephen M. Cordner, Isidro Cortés-Ciriano, Kyle Covington, Prue A. Cowin, Brian Craft, David Craft, Chad J. Creighton, Yupeng Cun, Erin Curley, Ioana Cutcutache, Karolina Czajka, Bogdan Czerniak, Rebecca A. Dagg, Ludmila Danilova, Maria Vittoria Davi, Natalie R. Davidson, Helen Davies, Ian J. Davis, Brandi N. Davis-Dusenbery, Kevin J. Dawson, Francisco M. De La Vega, Ricardo De Paoli-Iseppi, Timothy Defreitas, Angelo P. Dei Tos, Olivier Delaneau, John A. Demchok, Jonas Demeulemeester, German M. Demidov, Deniz Demircioğlu, Nening M. Dennis, Robert E. Denroche, Stefan C. Dentro, Nikita Desai, Vikram Deshpande, Amit G. Deshwar, Christine Desmedt, Jordi Deu-Pons, Noreen Dhalla, Neesha C. Dhani, Priyanka Dhingra, Rajiv Dhir, Anthony DiBiase, Klev Diamanti, Li Ding, Shuai Ding, Huy Q. Dinh, Luc Dirix, HarshaVardhan Doddapaneni, Nilgun Donmez, Michelle T. Dow, Ronny Drapkin, Oliver Drechsel, Ruben M. Drews, Serge Serge, Tim Dudderidge, Ana Dueso-Barroso, Andrew J. Dunford, Michael Dunn, Lewis Jonathan Dursi, Fraser R. Duthie, Ken Dutton-Regester, Jenna Eagles, Douglas F. Easton, Stuart Edmonds, Paul A. Edwards, Sandra E. Edwards, Rosalind A. Eeles, Anna Ehinger, Juergen Eils, Roland Eils, Adel El-Naggar, Matthew Eldridge, Kyle Ellrott, Serap Erkek, Georgia Escaramis, Shadrielle M. G. Espiritu, Xavier Estivill, Dariush Etemadmoghadam, Jorunn E. Eyfjord, Bishoy M. Faltas, Daiming Fan, Yu Fan, William C. Faquin, Claudiu Farcas, Matteo Fassan, Aquila Fatima, Francesco Favero, Nodirjon Fayzullaev, Ina Felau, Sian Fereday, Martin L. Ferguson, Vincent Ferretti, Lars Feuerbach, Matthew A. Field, J. Lynn Fink, Gaetano Finocchiaro, Cyril Fisher, Matthew W. Fittall, Anna Fitzgerald, Rebecca C. Fitzgerald, Adrienne M. Flanagan, Neil E. Fleshner, Paul Flicek, John A. Foekens, Kwun M. Fong, Nuno A. Fonseca, Christopher S. Foster, Natalie S. Fox, Michael Fraser, Scott Frazer, Milana Frenkel-Morgenstern, William Friedman, Joan Frigola, Catrina C. Fronick, Akihiro Fujimoto, Masashi Fujita, Masashi Fukayama, Lucinda A. Fulton, Robert S. Fulton, Mayuko Furuta, P. Andrew Futreal, Anja Füllgrabe, Stacey B. Gabriel, Steven Gallinger, Carlo Gambacorti-Passerini, Jianjiong Gao, Shengjie Gao, Levi Garraway, Øystein Garred, Erik Garrison, Dale W. Garsed, Nils Gehlenborg, Josep L. L. Gelpi, Joshy George, Daniela S. Gerhard, Clarissa Gerhauser, Jeffrey E. Gershenwald, Mark Gerstein, Moritz Gerstung, Gad Getz, Mohammed Ghori, Ronald Ghossein, Nasra H. Giama, Richard A. Gibbs, Bob Gibson, Anthony J. Gill, Pelvender Gill, Dilip D. Giri, Dominik Glodzik, Vincent J. Gnanapragasam, Maria Elisabeth Goebler, Mary J. Goldman, Carmen Gomez, Santiago Gonzalez, Abel Gonzalez-Perez, Dmitry A. Gordenin, James Gossage, Kunihito Gotoh, Ramaswamy Govindan, Dorthe Grabau, Janet S. Graham, Robert C. Grant, Anthony R. Green, Eric Green, Liliana Greger, Nicola Grehan, Sonia Grimaldi, Sean M. Grimmond, Robert L. Grossman, Adam Grundhoff, Gunes Gundem, Qianyun Guo, Manaswi Gupta, Shailja Gupta, Ivo G. Gut, Marta Gut, Jonathan Göke, Gavin Ha, Andrea Haake, David Haan, Siegfried Haas, Kerstin Haase, James E. Haber, Nina Habermann, Faraz Hach, Syed Haider, Natsuko Hama, Freddie C. Hamdy, Anne Hamilton, Mark P. Hamilton, Leng Han, George B. Hanna, Martin Hansmann, Nicholas J. Haradhvala, Olivier Harismendy, Ivon Harliwong, Arif O. Harmanci, Eoghan Harrington, Takanori Hasegawa, David Haussler, Steve Hawkins, Shinya Hayami, Shuto Hayashi, D. Neil Hayes, Stephen J. Hayes, Nicholas K. Hayward, Steven Hazell, Yao He, Allison P. Heath, Simon C. Heath, David Hedley, Apurva M. Hegde, David I. Heiman, Michael C. Heinold, Zachary Heins, Lawrence E. Heisler, Eva Hellstrom-Lindberg, Mohamed Helmy, Seong Gu Heo, Austin J. Hepperla, José María Heredia-Genestar, Carl Herrmann, Peter Hersey, Julian M. Hess, Holmfridur Hilmarsdottir, Jonathan Hinton, Satoshi Hirano, Nobuyoshi Hiraoka, Katherine A. Hoadley, Asger Hobolth, Ermin Hodzic, Jessica I. Hoell, Steve Hoffmann, Oliver Hofmann, Andrea Holbrook, Aliaksei Z. Holik, Michael A. Hollingsworth, Oliver Holmes, Robert A. Holt, Chen Hong, Eun Pyo Hong, Jongwhi H. Hong, Gerrit K. Hooijer, Henrik Hornshøj, Fumie Hosoda, Yong Hou, Volker Hovestadt, William Howat, Alan P. Hoyle, Ralph H. Hruban, Jianhong Hu, Taobo Hu, Xing Hua, Kuan-lin Huang, Mei Huang, Mi Ni Huang, Vincent Huang, Yi Huang, Wolfgang Huber, Thomas J. Hudson, Michael Hummel, Jillian A. Hung, David Huntsman, Ted R. Hupp, Jason Huse, Matthew R. Huska, Barbara Hutter, Carolyn M. Hutter, Daniel Hübschmann, Christine A. Iacobuzio-Donahue, Charles David Imbusch, Marcin Imielinski, Seiya Imoto, William B. Isaacs, Keren Isaev, Shumpei Ishikawa, Murat Iskar, S. M. Ashiqul Islam, Michael Ittmann, Sinisa Ivkovic, Jose M. G. Izarzugaza, Jocelyne Jacquemier, Valerie Jakrot, Nigel B. Jamieson, Gun Ho Jang, Se Jin Jang, Joy C. Jayaseelan, Reyka Jayasinghe, Stuart R. Jefferys, Karine Jegalian, Jennifer L. Jennings, Seung-Hyup Jeon, Lara Jerman, Yuan Ji, Wei Jiao, Peter A. Johansson, Amber L. Johns, Jeremy Johns, Rory Johnson, Todd A. Johnson, Clemency Jolly, Yann Joly, Jon G. Jonasson, Corbin D. Jones, David R. Jones, David T. W. Jones, Nic Jones, Steven J. M. Jones, Jos Jonkers, Young Seok Ju, Hartmut Juhl, Jongsun Jung, Malene Juul, Randi Istrup Juul, Sissel Juul, Natalie Jäger, Rolf Kabbe, Andre Kahles, Abdullah Kahraman, Vera B. Kaiser, Hojabr Kakavand, Sangeetha Kalimuthu, Christof von Kalle, Koo Jeong Kang, Katalin Karaszi, Beth Karlan, Rosa Karlić, Dennis Karsch, Katayoon Kasaian, Karin S. Kassahn, Hitoshi Katai, Mamoru Kato, Hiroto Katoh, Yoshiiku Kawakami, Jonathan D. Kay, Stephen H. Kazakoff, Marat D. Kazanov, Maria Keays, Electron Kebebew, Richard F. Kefford, Manolis Kellis, James G. Kench, Catherine J. Kennedy, Jules N. A. Kerssemakers, David Khoo, Vincent Khoo, Narong Khuntikeo, Ekta Khurana, Helena Kilpinen, Hark Kyun Kim, Hyung-Lae Kim, Hyung-Yong Kim, Hyunghwan Kim, Jaegil Kim, Jihoon Kim, Jong K. Kim, Youngwook Kim, Tari A. King, Wolfram Klapper, Kortine Kleinheinz, Leszek J. Klimczak, Stian Knappskog, Michael Kneba, Bartha M. Knoppers, Youngil Koh, Jan Komorowski, Daisuke Komura, Mitsuhiro Komura, Gu Kong, Marcel Kool, Jan O. Korbel, Viktoriya Korchina, Andrey Korshunov, Michael Koscher, Roelof Koster, Zsofia Kote-Jarai, Antonios Koures, Milena Kovacevic, Barbara Kremeyer, Helene Kretzmer, Markus Kreuz, Savitri Krishnamurthy, Dieter Kube, Kiran Kumar, Pardeep Kumar, Sushant Kumar, Yogesh Kumar, Ritika Kundra, Kirsten Kübler, Ralf Küppers, Jesper Lagergren, Phillip H. Lai, Peter W. Laird, Sunil R. Lakhani, Christopher M. Lalansingh, Emilie Lalonde, Fabien C. Lamaze, Adam Lambert, Eric Lander, Pablo Landgraf, Luca Landoni, Anita Langerød, Andrés Lanzós, Denis Larsimont, Erik Larsson, Mark Lathrop, Loretta M. S. Lau, Chris Lawerenz, Rita T. Lawlor, Michael S. Lawrence, Alexander J. Lazar, Ana Mijalkovic Lazic, Xuan Le, Darlene Lee, Donghoon Lee, Eunjung Alice Lee, Hee Jin Lee, Jake June-Koo Lee, Jeong-Yeon Lee, Juhee Lee, Ming Ta Michael Lee, Henry Lee-Six, Kjong-Van Lehmann, Hans Lehrach, Dido Lenze, Conrad R. Leonard, Daniel A. Leongamornlert, Ignaty Leshchiner, Louis Letourneau, Ivica Letunic, Douglas A. Levine, Lora Lewis, Tim Ley, Chang Li, Constance H. Li, Haiyan Irene Li, Jun Li, Lin Li, Shantao Li, Siliang Li, Xiaobo Li, Xiaotong Li, Xinyue Li, Yilong Li, Han Liang, Sheng-Ben Liang, Peter Lichter, Pei Lin, Ziao Lin, W. M. Linehan, Ole Christian Lingjærde, Dongbing Liu, Eric Minwei Liu, Fei-Fei Fei Liu, Fenglin Liu, Jia Liu, Xingmin Liu, Julie Livingstone, Dimitri Livitz, Naomi Livni, Lucas Lochovsky, Markus Loeffler, Georgina V. Long, Armando Lopez-Guillermo, Shaoke Lou, David N. Louis, Laurence B. Lovat, Yiling Lu, Yong-Jie Lu, Youyong Lu, Claudio Luchini, Ilinca Lungu, Xuemei Luo, Hayley J. Luxton, Andy G. Lynch, Lisa Lype, Cristina López, Carlos López-Otín, Eric Z. Ma, Yussanne Ma, Gaetan MacGrogan, Shona MacRae, Geoff Macintyre, Tobias Madsen, Kazuhiro Maejima, Andrea Mafficini, Dennis T. Maglinte, Arindam Maitra, Partha P. Majumder, Luca Malcovati, Salem Malikic, Giuseppe Malleo, Graham J. Mann, Luisa Mantovani-Löffler, Kathleen Marchal, Giovanni Marchegiani, Elaine R. Mardis, Adam A. Margolin, Maximillian G. Marin, Florian Markowetz, Julia Markowski, Jeffrey Marks, Tomas Marques-Bonet, Marco A. Marra, Luke Marsden, John W. M. Martens, Sancha Martin, Jose I. Martin-Subero, Iñigo Martincorena, Alexander Martinez-Fundichely, Yosef E. Maruvka, R. Jay Mashl, Charlie E. Massie, Thomas J. Matthew, Lucy Matthews, Erik Mayer, Simon Mayes, Michael Mayo, Faridah Mbabaali, Karen McCune, Ultan McDermott, Patrick D. McGillivray, Michael D. McLellan, John D. McPherson, John R. McPherson, Treasa A. McPherson, Samuel R. Meier, Alice Meng, Shaowu Meng, Andrew Menzies, Neil D. Merrett, Sue Merson, Matthew Meyerson, William Meyerson, Piotr A. Mieczkowski, George L. Mihaiescu, Sanja Mijalkovic, Tom Mikkelsen, Michele Milella, Linda Mileshkin, Christopher A. Miller, David K. Miller, Jessica K. Miller, Gordon B. Mills, Ana Milovanovic, Sarah Minner, Marco Miotto, Gisela Mir Arnau, Lisa Mirabello, Chris Mitchell, Thomas J. Mitchell, Satoru Miyano, Naoki Miyoshi, Shinichi Mizuno, Fruzsina Molnár-Gábor, Malcolm J. Moore, Richard A. Moore, Sandro Morganella, Quaid D. Morris, Carl Morrison, Lisle E. Mose, Catherine D. Moser, Ferran Muiños, Loris Mularoni, Andrew J. Mungall, Karen Mungall, Elizabeth A. Musgrove, Ville Mustonen, David Mutch, Francesc Muyas, Donna M. Muzny, Alfonso Muñoz, Jerome Myers, Ola Myklebost, Peter Möller, Genta Nagae, Adnan M. Nagrial, Hardeep K. Nahal-Bose, Hitoshi Nakagama, Hidewaki Nakagawa, Hiromi Nakamura, Toru Nakamura, Kaoru Nakano, Tannistha Nandi, Jyoti Nangalia, Mia Nastic, Arcadi Navarro, Fabio C. P. Navarro, David E. Neal, Gerd Nettekoven, Felicity Newell, Steven J. Newhouse, Yulia Newton, Alvin Wei Tian Ng, Anthony Ng, Jonathan Nicholson, David Nicol, Yongzhan Nie, G. Petur Nielsen, Morten Muhlig Nielsen, Serena Nik-Zainal, Michael S. Noble, Katia Nones, Paul A. Northcott, Faiyaz Notta, Brian D. O’Connor, Peter O’Donnell, Maria O’Donovan, Sarah O’Meara, Brian Patrick O’Neill, J. Robert O’Neill, David Ocana, Angelica Ochoa, Layla Oesper, Christopher Ogden, Hideki Ohdan, Kazuhiro Ohi, Lucila Ohno-Machado, Karin A. Oien, Akinyemi I. Ojesina, Hidenori Ojima, Takuji Okusaka, Larsson Omberg, Choon Kiat Ong, Stephan Ossowski, German Ott, B. F. Francis Ouellette, Christine P’ng, Marta Paczkowska, Salvatore Paiella, Chawalit Pairojkul, Marina Pajic, Qiang Pan-Hammarström, Elli Papaemmanuil, Irene Papatheodorou, Nagarajan Paramasivam, Ji Wan Park, Joong-Won Park, Keunchil Park, Kiejung Park, Peter J. Park, Joel S. Parker, Simon L. Parsons, Harvey Pass, Danielle Pasternack, Alessandro Pastore, Ann-Marie Patch, Iris Pauporté, Antonio Pea, John V. Pearson, Chandra Sekhar Pedamallu, Jakob Skou Pedersen, Paolo Pederzoli, Martin Peifer, Nathan A. Pennell, Charles M. Perou, Marc D. Perry, Gloria M. Petersen, Myron Peto, Nicholas Petrelli, Robert Petryszak, Stefan M. Pfister, Mark Phillips, Oriol Pich, Hilda A. Pickett, Todd D. Pihl, Nischalan Pillay, Sarah Pinder, Mark Pinese, Andreia V. Pinho, Esa Pitkänen, Xavier Pivot, Elena Piñeiro-Yáñez, Laura Planko, Christoph Plass, Paz Polak, Tirso Pons, Irinel Popescu, Olga Potapova, Aparna Prasad, Shaun R. Preston, Manuel Prinz, Antonia L. Pritchard, Stephenie D. Prokopec, Elena Provenzano, Xose S. Puente, Sonia Puig, Montserrat Puiggròs, Sergio Pulido-Tamayo, Gulietta M. Pupo, Colin A. Purdie, Michael C. Quinn, Raquel Rabionet, Janet S. Rader, Bernhard Radlwimmer, Petar Radovic, Benjamin Raeder, Keiran M. Raine, Manasa Ramakrishna, Kamna Ramakrishnan, Suresh Ramalingam, Benjamin J. Raphael, W. Kimryn Rathmell, Tobias Rausch, Guido Reifenberger, Jüri Reimand, Jorge Reis-Filho, Victor Reuter, Iker Reyes-Salazar, Matthew A. Reyna, Sheila M. Reynolds, Esther Rheinbay, Yasser Riazalhosseini, Andrea L. Richardson, Julia Richter, Matthew Ringel, Markus Ringnér, Yasushi Rino, Karsten Rippe, Jeffrey Roach, Lewis R. Roberts, Nicola D. Roberts, Steven A. Roberts, A. Gordon Robertson, Alan J. Robertson, Javier Bartolomé Rodriguez, Bernardo Rodriguez-Martin, F. Germán Rodríguez-González, Michael H. A. Roehrl, Marius Rohde, Hirofumi Rokutan, Gilles Romieu, Ilse Rooman, Tom Roques, Daniel Rosebrock, Mara Rosenberg, Philip C. Rosenstiel, Andreas Rosenwald, Edward W. Rowe, Romina Royo, Steven G. Rozen, Yulia Rubanova, Mark A. Rubin, Carlota Rubio-Perez, Vasilisa A. Rudneva, Borislav C. Rusev, Andrea Ruzzenente, Gunnar Rätsch, Radhakrishnan Sabarinathan, Veronica Y. Sabelnykova, Sara Sadeghi, S. Cenk Sahinalp, Natalie Saini, Mihoko Saito-Adachi, Gordon Saksena, Adriana Salcedo, Roberto Salgado, Leonidas Salichos, Richard Sallari, Charles Saller, Roberto Salvia, Michelle Sam, Jaswinder S. Samra, Francisco Sanchez-Vega, Chris Sander, Grant Sanders, Rajiv Sarin, Iman Sarrafi, Aya Sasaki-Oku, Torill Sauer, Guido Sauter, Robyn P. M. Saw, Maria Scardoni, Christopher J. Scarlett, Aldo Scarpa, Ghislaine Scelo, Dirk Schadendorf, Jacqueline E. Schein, Markus B. Schilhabel, Matthias Schlesner, Thorsten Schlomm, Heather K. Schmidt, Sarah-Jane Schramm, Stefan Schreiber, Nikolaus Schultz, Steven E. Schumacher, Roland F. Schwarz, Richard A. Scolyer, David Scott, Ralph Scully, Raja Seethala, Ayellet V. Segre, Iris Selander, Colin A. Semple, Yasin Senbabaoglu, Subhajit Sengupta, Elisabetta Sereni, Stefano Serra, Dennis C. Sgroi, Mark Shackleton, Nimish C. Shah, Sagedeh Shahabi, Catherine A. Shang, Ping Shang, Ofer Shapira, Troy Shelton, Ciyue Shen, Hui Shen, Rebecca Shepherd, Ruian Shi, Yan Shi, Yu-Jia Shiah, Tatsuhiro Shibata, Juliann Shih, Eigo Shimizu, Kiyo Shimizu, Seung Jun Shin, Yuichi Shiraishi, Tal Shmaya, Ilya Shmulevich, Solomon I. Shorser, Charles Short, Raunak Shrestha, Suyash S. Shringarpure, Craig Shriver, Shimin Shuai, Nikos Sidiropoulos, Reiner Siebert, Anieta M. Sieuwerts, Lina Sieverling, Sabina Signoretti, Katarzyna O. Sikora, Michele Simbolo, Ronald Simon, Janae V. Simons, Jared T. Simpson, Peter T. Simpson, Samuel Singer, Nasa Sinnott-Armstrong, Payal Sipahimalani, Tara J. Skelly, Marcel Smid, Jaclyn Smith, Karen Smith-McCune, Nicholas D. Socci, Heidi J. Sofia, Matthew G. Soloway, Lei Song, Anil K. Sood, Sharmila Sothi, Christos Sotiriou, Cameron M. Soulette, Paul N. Span, Paul T. Spellman, Nicola Sperandio, Andrew J. Spillane, Oliver Spiro, Jonathan Spring, Johan Staaf, Peter F. Stadler, Peter Staib, Stefan G. Stark, Lucy Stebbings, Ólafur Andri Stefánsson, Oliver Stegle, Lincoln D. Stein, Alasdair Stenhouse, Chip Stewart, Stephan Stilgenbauer, Miranda D. Stobbe, Michael R. Stratton, Jonathan R. Stretch, Adam J. Struck, Joshua M. Stuart, Henk G. Stunnenberg, Hong Su, Xiaoping Su, Ren X. Sun, Stephanie Sungalee, Hana Susak, Akihiro Suzuki, Fred Sweep, Monika Szczepanowski, Holger Sültmann, Takashi Yugawa, Angela Tam, David Tamborero, Benita Kiat Tee Tan, Donghui Tan, Patrick Tan, Hiroko Tanaka, Hirokazu Taniguchi, Tomas J. Tanskanen, Maxime Tarabichi, Roy Tarnuzzer, Patrick Tarpey, Morgan L. Taschuk, Kenji Tatsuno, Simon Tavaré, Darrin F. Taylor, Amaro Taylor-Weiner, Jon W. Teague, Bin Tean Teh, Varsha Tembe, Javier Temes, Kevin Thai, Sarah P. Thayer, Nina Thiessen, Gilles Thomas, Sarah Thomas, Alan Thompson, Alastair M. Thompson, John F. F. Thompson, R. Houston Thompson, Heather Thorne, Leigh B. Thorne, Adrian Thorogood, Grace Tiao, Nebojsa Tijanic, Lee E. Timms, Roberto Tirabosco, Marta Tojo, Stefania Tommasi, Christopher W. Toon, Umut H. Toprak, David Torrents, Giampaolo Tortora, Jörg Tost, Yasushi Totoki, David Townend, Nadia Traficante, Isabelle Treilleux, Jean-Rémi Trotta, Lorenz H. P. Trümper, Ming Tsao, Tatsuhiko Tsunoda, Jose M. C. Tubio, Olga Tucker, Richard Turkington, Daniel J. Turner, Andrew Tutt, Masaki Ueno, Naoto T. Ueno, Christopher Umbricht, Husen M. Umer, Timothy J. Underwood, Lara Urban, Tomoko Urushidate, Tetsuo Ushiku, Liis Uusküla-Reimand, Alfonso Valencia, David J. Van Den Berg, Steven Van Laere, Peter Van Loo, Erwin G. Van Meir, Gert G. Van den Eynden, Theodorus Van der Kwast, Naveen Vasudev, Miguel Vazquez, Ravikiran Vedururu, Umadevi Veluvolu, Shankar Vembu, Lieven P. C. Verbeke, Peter Vermeulen, Clare Verrill, Alain Viari, David Vicente, Caterina Vicentini, K. VijayRaghavan, Juris Viksna, Ricardo E. Vilain, Izar Villasante, Anne Vincent-Salomon, Tapio Visakorpi, Douglas Voet, Paresh Vyas, Ignacio Vázquez-García, Nick M. Waddell, Nicola Waddell, Claes Wadelius, Lina Wadi, Rabea Wagener, Jeremiah A. Wala, Jian Wang, Jiayin Wang, Linghua Wang, Qi Wang, Wenyi Wang, Yumeng Wang, Zhining Wang, Paul M. Waring, Hans-Jörg Warnatz, Jonathan Warrell, Anne Y. Warren, Sebastian M. Waszak, David C. Wedge, Dieter Weichenhan, Paul Weinberger, John N. Weinstein, Joachim Weischenfeldt, Daniel J. Weisenberger, Ian Welch, Michael C. Wendl, Johannes Werner, Justin P. Whalley, David A. Wheeler, Hayley C. Whitaker, Dennis Wigle, Matthew D. Wilkerson, Ashley Williams, James S. Wilmott, Gavin W. Wilson, Julie M. Wilson, Richard K. Wilson, Boris Winterhoff, Jeffrey A. Wintersinger, Maciej Wiznerowicz, Stephan Wolf, Bernice H. Wong, Tina Wong, Winghing Wong, Youngchoon Woo, Scott Wood, Bradly G. Wouters, Adam J. Wright, Derek W. Wright, Mark H. Wright, Chin-Lee Wu, Dai-Ying Wu, Guanming Wu, Jianmin Wu, Kui Wu, Yang Wu, Zhenggang Wu, Liu Xi, Tian Xia, Qian Xiang, Xiao Xiao, Rui Xing, Heng Xiong, Qinying Xu, Yanxun Xu, Hong Xue, Shinichi Yachida, Sergei Yakneen, Rui Yamaguchi, Takafumi N. Yamaguchi, Masakazu Yamamoto, Shogo Yamamoto, Hiroki Yamaue, Fan Yang, Huanming Yang, Jean Y. Yang, Liming Yang, Lixing Yang, Shanlin Yang, Tsun-Po Yang, Yang Yang, Xiaotong Yao, Marie-Laure Yaspo, Lucy Yates, Christina Yau, Chen Ye, Kai Ye, Venkata D. Yellapantula, Christopher J. Yoon, Sung-Soo Yoon, Fouad Yousif, Jun Yu, Kaixian Yu, Willie Yu, Yingyan Yu, Ke Yuan, Yuan Yuan, Denis Yuen, Christina K. Yung, Olga Zaikova, Jorge Zamora, Marc Zapatka, Jean C. Zenklusen, Thorsten Zenz, Nikolajs Zeps, Cheng-Zhong Zhang, Fan Zhang, Hailei Zhang, Hongwei Zhang, Hongxin Zhang, Jiashan Zhang, Jing Zhang, Junjun Zhang, Xiuqing Zhang, Xuanping Zhang, Yan Zhang, Zemin Zhang, Zhongming Zhao, Liangtao Zheng, Xiuqing Zheng, Wanding Zhou, Yong Zhou, Bin Zhu, Hongtu Zhu, Jingchun Zhu, Shida Zhu, Lihua Zou, Xueqing Zou, Anna deFazio, Nicholas van As, Carolien H. M. van Deurzen, Marc J. van de Vijver, L. van’t Veer, Christian von Mering

**Affiliations:** 1 Department of Molecular Genetics, University of Toronto, Toronto, ON Canada M5S 1A8; 2 Computational Biology Program, Ontario Institute for Cancer Research, Toronto, ON Canada M5G 0A3; 3 Division of General Surgery, Toronto General Hospital, Toronto, ON Canada M5G 2C4; 4 Lunenfeld-Tanenbaum Research Institute, Mount Sinai Hospital, Toronto, ON Canada M5G 1X5; 5 Wellcome Sanger Institute, Wellcome Genome Campus, Cambridge, CB10 1SA UK; 6 Department of Genomic Medicine, The University of Texas MD Anderson Cancer Center, Houston, TX 77030 USA; 7 Quantitative & Computational Biosciences Graduate Program, Baylor College of Medicine, Houston, TX 77030 USA; 8 The Jackson Laboratory for Genomic Medicine, Farmington, CT 06032 USA; 9 Broad Institute of MIT and Harvard, Cambridge, MA 02142 USA; 10 Dana-Farber/Boston Children’s Cancer and Blood Disorders Center, Boston, MA 02215 USA; 11 Department of Pediatrics, Harvard Medical School, Boston, MA 02115 USA; 12 Department of Medical Oncology, Dana-Farber Cancer Institute, Boston, MA 02115 USA; 13 Harvard Medical School, Boston, MA 02115 USA; 14 Department of Mathematics, Aarhus University, Aarhus, 8000 Denmark; 15 Department of Molecular Medicine (MOMA), Aarhus University Hospital, Aarhus, N 8200 Denmark; 16 Laboratory for Medical Science Mathematics, RIKEN Center for Integrative Medical Sciences, Yokohama, Kanagawa 230-0045 Japan; 17 RIKEN Center for Integrative Medical Sciences, Yokohama, Kanagawa 230-0045 Japan; 18 Technical University of Denmark, Lyngby, 2800 Denmark; 19 University of Copenhagen, Copenhagen, 2200 Denmark; 20 Department of Haematology, University of Cambridge, Cambridge, CB2 2XY UK; 21 Department for BioMedical Research, University of Bern, Bern, 3008 Switzerland; 22 Department of Medical Oncology, Inselspital, University Hospital and University of Bern, Bern, 3010 Switzerland; 23 Graduate School for Cellular and Biomedical Sciences, University of Bern, Bern, 3012 Switzerland; 24 Department of Genitourinary Medical Oncology - Research, Division of Cancer Medicine, The University of Texas MD Anderson Cancer Center, Houston, TX 77030 USA; 25 Department of Urology, Icahn School of Medicine at Mount Sinai, New York, NY 10029 USA; 26 Division of Theoretical Bioinformatics, German Cancer Research Center (DKFZ), Heidelberg, 69120 Germany; 27 Faculty of Biosciences, Heidelberg University, Heidelberg, 69120 Germany; 28 University of Texas MD Anderson Cancer Center, Houston, TX 77030 USA; 29 Korea Advanced Institute of Science and Technology, Daejeon, 34141 South Korea; 30 Institute for Research in Biomedicine (IRB Barcelona), The Barcelona Institute of Science and Technology, Barcelona, 8003 Spain; 31 Research Program on Biomedical Informatics, Universitat Pompeu Fabra, Barcelona, 08002 Spain; 32 Department of Physiology and Biophysics, Weill Cornell Medicine, New York, NY 10065 USA; 33 Institute for Computational Biomedicine, Weill Cornell Medicine, New York, NY 10021 USA; 34 Science for Life Laboratory, Department of Cell and Molecular Biology, Uppsala University, Uppsala, SE-75124 Sweden; 35 Division of Applied Bioinformatics, German Cancer Research Center (DKFZ), Heidelberg, 69120 Germany; 36 Barcelona Supercomputing Center (BSC), Barcelona, 08034 Spain; 37 Queensland Centre for Medical Genomics, Institute for Molecular Bioscience, The University of Queensland, Brisbane, QLD 4072 Australia; 38 CIBIO/InBIO - Research Center in Biodiversity and Genetic Resources, Universidade do Porto, Vairão, 4485-601 Portugal; 39 European Molecular Biology Laboratory, European Bioinformatics Institute (EMBL-EBI), Wellcome Genome Campus, Hinxton, Cambridge, CB10 1SD UK; 40 University of Milano Bicocca, Monza, 20052 Italy; 41 Peter MacCallum Cancer Centre, University of Melbourne, Melbourne, VIC 3000 Australia; 42 Sir Peter MacCallum Department of Oncology, Peter MacCallum Cancer Centre, University of Melbourne, Melbourne, VIC 3052 Australia; 43 Department of Computer Science, Yale University, New Haven, CT 06520 USA; 44 Department of Molecular Biophysics and Biochemistry, Yale University, New Haven, CT 06520 USA; 45 Program in Computational Biology and Bioinformatics, Yale University, New Haven, CT 06520 USA; 46 Center for Cancer Research, Massachusetts General Hospital, Boston, MA 02129 USA; 47 Department of Pathology, Massachusetts General Hospital, Boston, MA 02115 USA; 48 Bioinformatics Research Centre (BiRC), Aarhus University, Aarhus, 8000 Denmark; 49 CNAG-CRG, Centre for Genomic Regulation (CRG), Barcelona Institute of Science and Technology (BIST), Barcelona, 08028 Spain; 50 Universitat Pompeu Fabra (UPF), Barcelona, 08003 Spain; 51 Biomolecular Engineering Department, University of California Santa Cruz, Santa Cruz, CA 95064 USA; 52 Department of Internal Medicine, Stanford University, Stanford, CA 94305 USA; 53 Massachusetts General Hospital, Boston, MA 02114 USA; 54 Center for Precision Health, School of Biomedical Informatics, The University of Texas Health Science Center, Houston, TX 77030 USA; 55 The Donnelly Centre, University of Toronto, Toronto, ON M5S 3E1 Canada; 56 Health Data Science Unit, University Clinics, Heidelberg, 69120 Germany; 57 Institute of Pharmacy and Molecular Biotechnology and BioQuant, Heidelberg University, Heidelberg, 69120 Germany; 58 Massachusetts General Hospital Center for Cancer Research, Charlestown, MA 02129 USA; 59 Simon Fraser University, Burnaby, BC V5A 1S6 Canada; 60 Department of Medical Biophysics, University of Toronto, Toronto, ON M5S 1A8 Canada; 61 Computational Biology Center, Memorial Sloan Kettering Cancer Center, New York, NY 10065 USA; 62 Department of Biology, ETH Zurich, Zürich, 8093 Switzerland; 63 Department of Computer Science, ETH Zurich, Zurich, 8092 Switzerland; 64 SIB Swiss Institute of Bioinformatics, Lausanne, 1015 Switzerland; 65 University Hospital Zurich, Zurich, 8091 Switzerland; 66 Clinical Bioinformatics, Swiss Institute of Bioinformatics, Geneva, 1202 Switzerland; 67 Institute for Pathology and Molecular Pathology, University Hospital Zurich, Zurich, 8091 Switzerland; 68 Institute of Molecular Life Sciences, University of Zurich, Zurich, 8057 Switzerland; 69 MIT Computer Science and Artificial Intelligence Laboratory, Massachusetts Institute of Technology, Cambridge, MA 02139 USA; 70 Controlled Department and Institution, New York, NY 10065 USA; 71 Englander Institute for Precision Medicine, Weill Cornell Medicine, New York, NY 10065 USA; 72 Research Core Center, National Cancer Centre Korea, Goyang-si, 410-769 South Korea; 73 Department of Health Sciences and Technology, Sungkyunkwan University School of Medicine, Seoul, 06351 South Korea; 74 Samsung Genome Institute, Samsung Medical Center, Seoul, Korea; 75 Institute of Computer Science, Polish Academy of Sciences, Warsawa, 01-248 Poland; 76 Genome Biology Unit, European Molecular Biology Laboratory (EMBL), Heidelberg, 69117 Germany; 77 Institute of Biomedicine, Sahlgrenska Academy at University of Gothenburg, Gothenburg, Sweden; 78 Department of Biology, ETH Zurich, Wolfgang-Pauli-Strasse 27, 8093 Zürich, Switzerland; 79 Harvard University, Cambridge, MA 02138 USA; 80 Memorial Sloan Kettering Cancer Center, New York, NY 10065 USA; 81 Department of Information Technology, Ghent University, Ghent, B-9000 Belgium; 82 Department of Plant Biotechnology and Bioinformatics, Ghent University, Ghent, B-9000 Belgium; 83 Yale School of Medicine, Yale University, New Haven, CT 06520 USA; 84 Institute for Research in Biomedicine (IRB Barcelona), The Barcelona Institute of Science and Technology, Barcelona, Spain; 85 Division of Hematology-Oncology, Samsung Medical Center, Sungkyunkwan University School of Medicine, Seoul, 06351 South Korea; 86 Samsung Advanced Institute for Health Sciences and Technology, Sungkyunkwan University School of Medicine, Seoul, 06351 South Korea; 87 Cheonan Industry-Academic Collaboration Foundation, Sangmyung University, Cheonan, 31066 South Korea; 88 Spanish National Cancer Research Centre, Madrid, 28029 Spain; 89 Department of Computer Science, Princeton University, Princeton, NJ 08540 USA; 90 Bern Center for Precision Medicine, University Hospital of Bern, University of Bern, Bern, 3008 Switzerland; 91 Englander Institute for Precision Medicine, Weill Cornell Medicine and New York Presbyterian Hospital, New York, NY 10021 USA; 92 Meyer Cancer Center, Weill Cornell Medicine, New York, NY 10065 USA; 93 Pathology and Laboratory, Weill Cornell Medical College, New York, NY 10021 USA; 94 Vall d’Hebron Institute of Oncology: VHIO, Barcelona, 08035 Spain; 95 Indiana University, Bloomington, IN 47405 USA; 96 Vancouver Prostate Centre, Vancouver, BC V6H 3Z6 Canada; 97 cBio Center, Dana-Farber Cancer Institute, Harvard Medical School, Boston, MA 02115 USA; 98 Department of Cell Biology, Harvard Medical School, Boston, MA 02115 USA; 99 Department of Cancer Biology, Dana-Farber Cancer Institute, Boston, MA 02215 USA; 100 cBio Center, Dana-Farber Cancer Institute, Boston, MA 02215 USA; 101 Finsen Laboratory and Biotech Research & Innovation Centre (BRIC), University of Copenhagen, Copenhagen, 2200 Denmark; 102 Department of Genetics, Stanford University School of Medicine, Stanford, CA 94305 USA; 103 CREST, Japan Science and Technology Agency, Tokyo, 113-0033 Japan; 104 Department of Medical Science Mathematics, Medical Research Institute, Tokyo Medical and Dental University, Bunkyo-ku Tokyo, 113-8510 Japan; 105 Laboratory for Medical Science Mathematics, Department of Biological Sciences, Graduate School of Science, The University of Tokyo, Bunkyo-ku Tokyo, 113-0033 Japan; 106 Department of Oncology-Pathology, Science for Life Laboratory, Karolinska Institute, Stockholm, Sweden; 107 Department of Gene Technology, Tallinn University of Technology, Tallinn, 12616 Estonia; 108 Genetics & Genome Biology Program, SickKids Research Institute, The Hospital for Sick Children, Toronto, ON M5G 1X8 Canada; 109 Institució Catalana de Recerca i Estudis Avançats (ICREA), Barcelona, 08010 Spain; 110 Department of Clinical and Molecular Medicine, Faculty of Medicine and Health Sciences, Norwegian University of Science and Technology, Trondheim, 7030 Norway; 111 Department of Information Technology, Ghent University, Interuniversitair Micro-Electronica Centrum (IMEC), Ghent, B-9000 Belgium; 112 Science for Life Laboratory, Department of Immunology, Genetics and Pathology, Uppsala University, Uppsala, SE-75108 Sweden; 113 School of Computer Science and Technology, Xi’an Jiaotong University, Xi’an, 710048 China; 114 School of Electronic and Information Engineering, Xi’an Jiaotong University, Xi’an, 710048 China; 115 The McDonnell Genome Institute at Washington University, St Louis, MO 63108 USA; 116 Department of Urology, Charité Universitätsmedizin Berlin, Berlin, 10117 Germany; 117 Department of Molecular and Human Genetics, Baylor College of Medicine, Houston, TX 77030 USA; 118 Human Genome Sequencing Center, Baylor College of Medicine, Houston, TX 77030 USA; 119 Oregon Health & Sciences University, Portland, OR 97239 USA; 120 Department of Medicine and Therapeutics, The Chinese University of Hong Kong, Shatin, NT Hong Kong, China; 121 The University of Texas Health Science Center at Houston, Houston, TX 77030 USA; 122 Department of Biomedical Informatics, College of Medicine, The Ohio State University, Columbus, OH 43210 USA; 123 The Ohio State University Comprehensive Cancer Center (OSUCCC—James), Columbus, OH 43210 USA; 124 The University of Texas School of Biomedical Informatics (SBMI) at Houston, Houston, TX 77030 USA; 125 Department of Biochemistry and Molecular Genetics, Feinberg School of Medicine, Northwestern University, Chicago, IL 60637 USA; 126 Institute of Molecular Life Sciences and Swiss Institute of Bioinformatics, University of Zurich, Zurich, 8057 Switzerland; 200 Applied Tumor Genomics Research Program, Research Programs Unit, University of Helsinki, Helsinki, Finland; 201 Wellcome Sanger Institute, Wellcome Genome Campus, Hinxton, UK; 202 Memorial Sloan Kettering Cancer Center, New York, NY USA; 203 Genome Science Division, Research Center for Advanced Science and Technology, University of Tokyo, Tokyo, Japan; 204 Department of Surgery, University of Chicago, Chicago, IL USA; 205 Department of Surgery, Division of Hepatobiliary and Pancreatic Surgery, School of Medicine, Keimyung University Dongsan Medical Center, Daegu, South Korea; 206 Department of Oncology, Gil Medical Center, Gachon University, Incheon, South Korea; 207 Hiroshima University, Hiroshima, Japan; 208 Department of Bioinformatics and Computational Biology, The University of Texas MD Anderson Cancer Center, Houston, TX USA; 209 University of Texas MD Anderson Cancer Center, Houston, TX USA; 210 King Faisal Specialist Hospital and Research Centre, Al Maather, Riyadh, Saudi Arabia; 211 Bioinformatics Unit, Spanish National Cancer Research Centre (CNIO), Madrid, Spain; 212 Bioinformatics Core Facility, University Medical Center Hamburg, Hamburg, Germany; 213 Heinrich Pette Institute, Leibniz Institute for Experimental Virology, Hamburg, Germany; 214 Ontario Tumour Bank, Ontario Institute for Cancer Research, Toronto, ON Canada; 215 Department of Pathology, The University of Texas MD Anderson Cancer Center, Houston, TX USA; 216 Laboratory of Pathology, Center for Cancer Research, National Cancer Institute, Bethesda, MD USA; 217 Department of Cellular and Molecular Medicine and Department of Bioengineering, University of California San Diego, La Jolla, CA USA; 218 UC San Diego Moores Cancer Center, San Diego, CA USA; 219 Canada’s Michael Smith Genome Sciences Centre, BC Cancer, Vancouver, BC Canada; 220 Sir Peter MacCallum Department of Oncology, Peter MacCallum Cancer Centre, University of Melbourne, Melbourne, VIC Australia; 221 Centre for Research in Molecular Medicine and Chronic Diseases (CiMUS), Universidade de Santiago de Compostela, Santiago de Compostela, Spain; 222 Department of Zoology, Genetics and Physical Anthropology, (CiMUS), Universidade de Santiago de Compostela, Santiago de Compostela, Spain; 223 The Biomedical Research Centre (CINBIO), Universidade de Vigo, Vigo, Spain; 224 Royal National Orthopaedic Hospital - Bolsover, London, UK; 225 Department of Genomic Medicine, The University of Texas MD Anderson Cancer Center, Houston, TX USA; 226 Quantitative and Computational Biosciences Graduate Program, Baylor College of Medicine, Houston, TX USA; 227 The Jackson Laboratory for Genomic Medicine, Farmington, CT USA; 228 Genome Informatics Program, Ontario Institute for Cancer Research, Toronto, ON Canada; 229 Institute of Human Genetics, Christian-Albrechts-University, Kiel, Germany; 230 Institute of Human Genetics, Ulm University and Ulm University Medical Center, Ulm, Germany; 231 Queensland Centre for Medical Genomics, Institute for Molecular Bioscience, University of Queensland, St. Lucia, Brisbane, QLD Australia; 232 Salford Royal NHS Foundation Trust, Salford, UK; 233 Department of Surgery, Pancreas Institute, University and Hospital Trust of Verona, Verona, Italy; 234 Molecular and Medical Genetics, OHSU Knight Cancer Institute, Oregon Health and Science University, Portland, OR USA; 235 Department of Molecular Oncology, BC Cancer Research Centre, Vancouver, BC Canada; 236 The McDonnell Genome Institute at Washington University, St. Louis, MO USA; 237 University College London, London, UK; 238 Division of Cancer Genomics, National Cancer Center Research Institute, National Cancer Center, Tokyo, Japan; 239 DLR Project Management Agency, Bonn, Germany; 240 Tokyo Women’s Medical University, Tokyo, Japan; 241 Center for Molecular Oncology, Memorial Sloan Kettering Cancer Center, New York, NY USA; 242 Los Alamos National Laboratory, Los Alamos, NM USA; 243 Department of Pathology, University Health Network, Toronto General Hospital, Toronto, ON Canada; 244 Nottingham University Hospitals NHS Trust, Nottingham, UK; 245 Epigenomics and Cancer Risk Factors, German Cancer Research Center (DKFZ), Heidelberg, Germany; 246 Computational Biology Program, Ontario Institute for Cancer Research, Toronto, ON Canada; 247 Department of Molecular Genetics, University of Toronto, Toronto, ON Canada; 248 Vector Institute, Toronto, ON Canada; 249 Hematopathology Section, Institute of Pathology, Christian-Albrechts-University, Kiel, Germany; 250 Department of Pathology and Laboratory Medicine, School of Medicine, University of North Carolina at Chapel Hill, Chapel Hill, NC USA; 251 Department of Cancer Genetics, Institute for Cancer Research, Oslo University Hospital, The Norwegian Radium Hospital, Oslo, Norway; 252 Pathology, Hospital Clinic, Institut d’Investigacions Biomèdiques August Pi i Sunyer (IDIBAPS), University of Barcelona, Barcelona, Spain; 253 Department of Veterinary Medicine, Transmissible Cancer Group, University of Cambridge, Cambridge, UK; 254 Alvin J. Siteman Cancer Center, Washington University School of Medicine, St. Louis, MO USA; 255 Wolfson Wohl Cancer Research Centre, Institute of Cancer Sciences, University of Glasgow, Glasgow, UK; 256 Lineberger Comprehensive Cancer Center, University of North Carolina at Chapel Hill, Chapel Hill, NC USA; 257 Broad Institute of MIT and Harvard, Cambridge, MA USA; 258 Dana-Farber/Boston Children’s Cancer and Blood Disorders Center, Boston, MA USA; 259 Department of Pediatrics, Harvard Medical School, Boston, MA USA; 260 Leeds Institute of Medical Research @ St. James’s, University of Leeds, St. James’s University Hospital, Leeds, UK; 261 Department of Pathology and Diagnostics, University and Hospital Trust of Verona, Verona, Italy; 262 Department of Surgery, Princess Alexandra Hospital, Brisbane, QLD Australia; 263 Surgical Oncology Group, Diamantina Institute, University of Queensland, Brisbane, QLD Australia; 264 Department of Population and Quantitative Health Sciences, Case Western Reserve University School of Medicine, Cleveland, OH USA; 265 Research Health Analytics and Informatics, University Hospitals Cleveland Medical Center, Cleveland, OH USA; 266 Gloucester Royal Hospital, Gloucester, UK; 267 European Molecular Biology Laboratory, European Bioinformatics Institute (EMBL-EBI), Cambridge, UK; 268 Diagnostic Development, Ontario Institute for Cancer Research, Toronto, ON Canada; 269 Barcelona Supercomputing Center (BSC), Barcelona, Spain; 270 Arnie Charbonneau Cancer Institute, University of Calgary, Calgary, AB Canada; 271 Departments of Surgery and Oncology, University of Calgary, Calgary, AB Canada; 272 Department of Pathology, Oslo University Hospital, The Norwegian Radium Hospital, Oslo, Norway; 273 PanCuRx Translational Research Initiative, Ontario Institute for Cancer Research, Toronto, ON Canada; 274 Department of Oncology, Sidney Kimmel Comprehensive Cancer Center at Johns Hopkins University School of Medicine, Baltimore, MD USA; 275 University Hospital Southampton NHS Foundation Trust, Southampton, UK; 276 Royal Stoke University Hospital, Stoke-on-Trent, UK; 277 Genome Sequence Informatics, Ontario Institute for Cancer Research, Toronto, ON Canada; 278 Human Longevity Inc, San Diego, CA USA; 279 Olivia Newton-John Cancer Research Institute, La Trobe University, Heidelberg, VIC Australia; 280 Computer Network Information Center, Chinese Academy of Sciences, Beijing, China; 281 Genome Canada, Ottawa, ON Canada; 282 CNAG-CRG, Centre for Genomic Regulation (CRG), Barcelona Institute of Science and Technology (BIST), Barcelona, Spain; 283 Universitat Pompeu Fabra (UPF), Barcelona, Spain; 284 Buck Institute for Research on Aging, Novato, CA USA; 285 Duke University Medical Center, Durham, NC USA; 286 Department of Human Genetics, Hannover Medical School, Hannover, Germany; 287 Center for Bioinformatics and Functional Genomics, Cedars-Sinai Medical Center, Los Angeles, CA USA; 288 Department of Biomedical Sciences, Cedars-Sinai Medical Center, Los Angeles, CA USA; 289 The Hebrew University Faculty of Medicine, Jerusalem, Israel; 290 Barts Cancer Institute, Barts and the London School of Medicine and Dentistry, Queen Mary University of London, London, UK; 291 Department of Computer Science, Bioinformatics Group, University of Leipzig, Leipzig, Germany; 292 Interdisciplinary Center for Bioinformatics, University of Leipzig, Leipzig, Germany; 293 Transcriptome Bioinformatics, LIFE Research Center for Civilization Diseases, University of Leipzig, Leipzig, Germany; 294 Department of Medical Oncology, Dana-Farber Cancer Institute, Boston, MA USA; 295 Department of Cancer Biology, Dana-Farber Cancer Institute, Boston, MA USA; 296 Harvard Medical School, Boston, MA USA; 297 USC Norris Comprehensive Cancer Center, University of Southern California, Los Angeles, CA USA; 298 Department of Diagnostics and Public Health, University and Hospital Trust of Verona, Verona, Italy; 299 Department of Mathematics, Aarhus University, Aarhus, Denmark; 300 Department of Molecular Medicine (MOMA), Aarhus University Hospital, Aarhus N, Denmark; 301 Instituto Carlos Slim de la Salud, Mexico City, Mexico; 302 Department of Medical Biophysics, University of Toronto, Toronto, ON Canada; 303 Cancer Division, Garvan Institute of Medical Research, Kinghorn Cancer Centre, University of New South Wales (UNSW Sydney), Sydney, NSW Australia; 304 South Western Sydney Clinical School, Faculty of Medicine, University of New South Wales (UNSW Sydney), Liverpool, NSW Australia; 305 West of Scotland Pancreatic Unit, Glasgow Royal Infirmary, Glasgow, UK; 306 Center for Digital Health, Berlin Institute of Health and Charitè - Universitätsmedizin Berlin, Berlin, Germany; 307 Heidelberg Center for Personalized Oncology (DKFZ-HIPO), German Cancer Research Center (DKFZ), Heidelberg, Germany; 308 The Preston Robert Tisch Brain Tumor Center, Duke University Medical Center, Durham, NC USA; 309 Massachusetts General Hospital, Boston, MA USA; 310 National Institute of Biomedical Genomics, Kalyani, West Bengal India; 311 Institute of Clinical Medicine and Institute of Oral Biology, University of Oslo, Oslo, Norway; 312 University of North Carolina at Chapel Hill, Chapel Hill, NC USA; 313 ARC-Net Centre for Applied Research on Cancer, University and Hospital Trust of Verona, Verona, Italy; 314 The Institute of Cancer Research, London, UK; 315 Centre for Computational Biology, Duke-NUS Medical School, Singapore, Singapore; 316 Programme in Cancer and Stem Cell Biology, Duke-NUS Medical School, Singapore, Singapore; 317 Division of Oncology and Pathology, Department of Clinical Sciences Lund, Lund University, Lund, Sweden; 318 Department of Pediatric Oncology, Hematology and Clinical Immunology, Heinrich-Heine-University, Düsseldorf, Germany; 319 Laboratory for Medical Science Mathematics, RIKEN Center for Integrative Medical Sciences, Yokohama, Japan; 320 RIKEN Center for Integrative Medical Sciences, Yokohama, Japan; 321 Department of Internal Medicine/Hematology, Friedrich-Ebert-Hospital, Neumünster, Germany; 322 Departments of Dermatology and Pathology, Yale University, New Haven, CT USA; 323 Centre for Genomic Regulation (CRG), The Barcelona Institute of Science and Technology, Barcelona, Spain; 324 Radcliffe Department of Medicine, University of Oxford, Oxford, UK; 325 Canadian Center for Computational Genomics, McGill University, Montreal, QC Canada; 326 Department of Human Genetics, McGill University, Montreal, QC Canada; 327 Department of Human Genetics, University of California Los Angeles, Los Angeles, CA USA; 328 Department of Pharmacology, University of Toronto, Toronto, ON Canada; 329 Faculty of Medicine and Health Technology, Tampere University and Tays Cancer Center, Tampere University Hospital, Tampere, Finland; 330 Haematology, Leeds Teaching Hospitals NHS Trust, Leeds, UK; 331 Translational Research and Innovation, Centre Léon Bérard, Lyon, France; 332 Fox Chase Cancer Center, Philadelphia, PA USA; 333 International Agency for Research on Cancer, World Health Organization, Lyon, France; 334 Earlham Institute, Norwich, UK; 335 Norwich Medical School, University of East Anglia, Norwich, UK; 336 Department of Molecular Biology, Faculty of Science, Radboud Institute for Molecular Life Sciences, Radboud University, Nijmegen, HB The Netherlands; 337 CRUK Manchester Institute and Centre, Manchester, UK; 338 Department of Radiation Oncology, University of Toronto, Toronto, ON Canada; 339 Division of Cancer Sciences, Manchester Cancer Research Centre, University of Manchester, Manchester, UK; 340 Radiation Medicine Program, Princess Margaret Cancer Centre, Toronto, ON Canada; 341 Department of Pathology, Brigham and Women’s Hospital, Harvard Medical School, Boston, MA USA; 342 Department of Surgery, Division of Thoracic Surgery, The Johns Hopkins University School of Medicine, Baltimore, MD USA; 343 Division of Molecular Pathology, The Netherlands Cancer Institute, Oncode Institute, Amsterdam, CX The Netherlands; 344 Department of Biomolecular Engineering, University of California Santa Cruz, Santa Cruz, CA USA; 345 UC Santa Cruz Genomics Institute, University of California Santa Cruz, Santa Cruz, CA USA; 346 Division of Applied Bioinformatics, German Cancer Research Center (DKFZ), Heidelberg, Germany; 347 German Cancer Consortium (DKTK), German Cancer Research Center (DKFZ), Heidelberg, Germany; 348 National Center for Tumor Diseases (NCT) Heidelberg, Heidelberg, Germany; 349 Center for Biological Sequence Analysis, Department of Bio and Health Informatics, Technical University of Denmark, Lyngby, Denmark; 350 Novo Nordisk Foundation Center for Protein Research, University of Copenhagen, Copenhagen, Denmark; 351 Institute for Molecular Bioscience, University of Queensland, St. Lucia, Brisbane, QLD Australia; 352 Biomedical Engineering, Oregon Health and Science University, Portland, OR USA; 353 Division of Theoretical Bioinformatics, German Cancer Research Center (DKFZ), Heidelberg, Germany; 354 Institute of Pharmacy and Molecular Biotechnology and BioQuant, Heidelberg University, Heidelberg, Germany; 355 Federal Ministry of Education and Research, Berlin, Germany; 356 Melanoma Institute Australia, University of Sydney, Sydney, NSW Australia; 357 Pediatric Hematology and Oncology, University Hospital Muenster, Muenster, Germany; 358 Department of Pathology, Johns Hopkins University School of Medicine, Baltimore, MD USA; 359 McKusick-Nathans Institute of Genetic Medicine, Sidney Kimmel Comprehensive Cancer Center at Johns Hopkins University School of Medicine, Baltimore, MD USA; 360 Foundation Medicine, Inc, Cambridge, MA USA; 361 Department of Biomedical Data Science, Stanford University School of Medicine, Stanford, CA USA; 362 Department of Genetics, Stanford University School of Medicine, Stanford, CA USA; 363 Bakar Computational Health Sciences Institute and Department of Pediatrics, University of California, San Francisco, CA USA; 364 Institute of Clinical Medicine, Faculty of Medicine, University of Oslo, Oslo, Norway; 365 National Cancer Institute, National Institutes of Health, Bethesda, MD USA; 366 Royal Marsden NHS Foundation Trust, London and Sutton, UK; 367 Genome Biology Unit, European Molecular Biology Laboratory (EMBL), Heidelberg, Germany; 368 Department of Oncology, University of Cambridge, Cambridge, UK; 369 Li Ka Shing Centre, Cancer Research UK Cambridge Institute, University of Cambridge, Cambridge, UK; 370 Institut Gustave Roussy, Villejuif, France; 371 Cambridge University Hospitals NHS Foundation Trust, Cambridge, UK; 372 Department of Haematology, University of Cambridge, Cambridge, UK; 373 Anatomia Patológica, Hospital Clinic, Institut d’Investigacions Biomèdiques August Pi i Sunyer (IDIBAPS), University of Barcelona, Barcelona, Spain; 374 Spanish Ministry of Science and Innovation, Madrid, Spain; 375 University of Michigan Comprehensive Cancer Center, Ann Arbor, MI USA; 376 Department for BioMedical Research, University of Bern, Bern, Switzerland; 377 Department of Medical Oncology, Inselspital, University Hospital and University of Bern, Bern, Switzerland; 378 Graduate School for Cellular and Biomedical Sciences, University of Bern, Bern, Switzerland; 379 University of Pavia, Pavia, Italy; 380 University of Alabama at Birmingham, Birmingham, AL USA; 381 UHN Program in BioSpecimen Sciences, Toronto General Hospital, Toronto, ON Canada; 382 Department of Urology, Icahn School of Medicine at Mount Sinai, New York, NY USA; 383 Centre for Law and Genetics, University of Tasmania, Sandy Bay Campus, Hobart, TAS Australia; 384 Faculty of Biosciences, Heidelberg University, Heidelberg, Germany; 385 Department of Biochemistry, Microbiology and Immunology, Faculty of Medicine, University of Ottawa, Ottawa, ON Canada; 386 Division of Anatomic Pathology, Mayo Clinic, Rochester, MN USA; 387 Division of Cancer Epidemiology and Genetics, National Cancer Institute, National Institutes of Health, Bethesda, MD USA; 388 Illawarra Shoalhaven Local Health District L3 Illawarra Cancer Care Centre, Wollongong Hospital, Wollongong, NSW Australia; 389 BioForA, French National Institute for Agriculture, Food, and Environment (INRAE), ONF, Orléans, France; 390 Department of Biostatistics, Bloomberg School of Public Health, Johns Hopkins University, Baltimore, MD USA; 391 University of California San Diego, San Diego, CA USA; 392 Division of Experimental Pathology, Mayo Clinic, Rochester, MN USA; 393 Centre for Cancer Research, The Westmead Institute for Medical Research, University of Sydney, Sydney, NSW Australia; 394 Department of Gynaecological Oncology, Westmead Hospital, Sydney, NSW Australia; 395 PDXen Biosystems Inc, Seoul, South Korea; 396 Korea Advanced Institute of Science and Technology, Daejeon, South Korea; 397 Electronics and Telecommunications Research Institute, Daejeon, South Korea; 398 Institut National du Cancer (INCA), Boulogne-Billancourt, France; 399 Department of Genetics, Informatics Institute, University of Alabama at Birmingham, Birmingham, AL USA; 400 Division of Medical Oncology, National Cancer Centre, Singapore, Singapore; 401 Medical Oncology, University and Hospital Trust of Verona, Verona, Italy; 402 Department of Pediatrics, University Hospital Schleswig-Holstein, Kiel, Germany; 403 Hepatobiliary/Pancreatic Surgical Oncology Program, University Health Network, Toronto, ON Canada; 404 School of Biological Sciences, University of Auckland, Auckland, New Zealand; 405 Department of Surgery, University of Melbourne, Parkville, VIC Australia; 406 The Murdoch Children’s Research Institute, Royal Children’s Hospital, Parkville, VIC Australia; 407 Walter and Eliza Hall Institute, Parkville, VIC Australia; 408 Vancouver Prostate Centre, Vancouver, Canada; 409 Lunenfeld-Tanenbaum Research Institute, Mount Sinai Hospital, Toronto, ON Canada; 410 University of East Anglia, Norwich, UK; 411 Norfolk and Norwich University Hospital NHS Trust, Norwich, UK; 412 Victorian Institute of Forensic Medicine, Southbank, VIC Australia; 413 Department of Biomedical Informatics, Harvard Medical School, Boston, MA USA; 414 Department of Chemistry, Centre for Molecular Science Informatics, University of Cambridge, Cambridge, UK; 415 Ludwig Center at Harvard Medical School, Boston, MA USA; 416 Human Genome Sequencing Center, Baylor College of Medicine, Houston, TX USA; 417 Peter MacCallum Cancer Centre, University of Melbourne, Melbourne, VIC Australia; 418 Physics Division, Optimization and Systems Biology Lab, Massachusetts General Hospital, Boston, MA USA; 419 Department of Medicine, Baylor College of Medicine, Houston, TX USA; 420 University of Cologne, Cologne, Germany; 421 International Genomics Consortium, Phoenix, AZ USA; 422 Genomics Research Program, Ontario Institute for Cancer Research, Toronto, ON Canada; 423 Barking Havering and Redbridge University Hospitals NHS Trust, Romford, UK; 424 Children’s Hospital at Westmead, University of Sydney, Sydney, NSW Australia; 425 Department of Medicine, Section of Endocrinology, University and Hospital Trust of Verona, Verona, Italy; 426 Computational Biology Center, Memorial Sloan Kettering Cancer Center, New York, NY USA; 427 Department of Biology, ETH Zurich, Zürich, Switzerland; 428 Department of Computer Science, ETH Zurich, Zurich, Switzerland; 429 SIB Swiss Institute of Bioinformatics, Lausanne, Switzerland; 430 Weill Cornell Medical College, New York, NY USA; 431 Academic Department of Medical Genetics, University of Cambridge, Addenbrooke’s Hospital, Cambridge, UK; 432 MRC Cancer Unit, University of Cambridge, Cambridge, UK; 433 Departments of Pediatrics and Genetics, University of North Carolina at Chapel Hill, Chapel Hill, NC USA; 434 Seven Bridges Genomics, Charlestown, MA USA; 435 Annai Systems, Inc, Carlsbad, CA USA; 436 Department of Pathology, General Hospital of Treviso, Department of Medicine, University of Padua, Treviso, Italy; 437 Department of Computational Biology, University of Lausanne, Lausanne, Switzerland; 438 Department of Genetic Medicine and Development, University of Geneva Medical School, Geneva, CH Switzerland; 439 Swiss Institute of Bioinformatics, University of Geneva, Geneva, CH Switzerland; 440 The Francis Crick Institute, London, UK; 441 University of Leuven, Leuven, Belgium; 442 Institute of Medical Genetics and Applied Genomics, University of Tübingen, Tübingen, Germany; 443 Computational and Systems Biology, Genome Institute of Singapore, Singapore, Singapore; 444 School of Computing, National University of Singapore, Singapore, Singapore; 445 Big Data Institute, Li Ka Shing Centre, University of Oxford, Oxford, UK; 446 Biomedical Data Science Laboratory, Francis Crick Institute, London, UK; 447 Bioinformatics Group, Department of Computer Science, University College London, London, UK; 448 The Edward S. Rogers Sr. Department of Electrical and Computer Engineering, University of Toronto, Toronto, ON Canada; 449 Breast Cancer Translational Research Laboratory JC Heuson, Institut Jules Bordet, Brussels, Belgium; 450 Department of Oncology, Laboratory for Translational Breast Cancer Research, KU Leuven, Leuven, Belgium; 451 Institute for Research in Biomedicine (IRB Barcelona), The Barcelona Institute of Science and Technology, Barcelona, Spain; 452 Research Program on Biomedical Informatics, Universitat Pompeu Fabra, Barcelona, Spain; 453 Division of Medical Oncology, Princess Margaret Cancer Centre, Toronto, ON Canada; 454 Department of Physiology and Biophysics, Weill Cornell Medicine, New York, NY USA; 455 Institute for Computational Biomedicine, Weill Cornell Medicine, New York, NY USA; 456 Department of Pathology, UPMC Shadyside, Pittsburgh, PA USA; 457 Independent Consultant, Wellesley, USA; 458 Department of Cell and Molecular Biology, Science for Life Laboratory, Uppsala University, Uppsala, Sweden; 459 Department of Medicine and Department of Genetics, Washington University School of Medicine, St. Louis, St. Louis, MO USA; 460 Hefei University of Technology, Anhui, China; 461 Translational Cancer Research Unit, GZA Hospitals St.-Augustinus, Center for Oncological Research, Faculty of Medicine and Health Sciences, University of Antwerp, Antwerp, Belgium; 462 Simon Fraser University, Burnaby, BC Canada; 463 University of Pennsylvania, Philadelphia, PA USA; 464 Faculty of Science and Technology, University of Vic—Central University of Catalonia (UVic-UCC), Vic, Spain; 465 The Wellcome Trust, London, UK; 466 The Hospital for Sick Children, Toronto, ON Canada; 467 Department of Pathology, Queen Elizabeth University Hospital, Glasgow, UK; 468 Department of Genetics and Computational Biology, QIMR Berghofer Medical Research Institute, Brisbane, QLD Australia; 469 Department of Oncology, Centre for Cancer Genetic Epidemiology, University of Cambridge, Cambridge, UK; 470 Department of Public Health and Primary Care, Centre for Cancer Genetic Epidemiology, University of Cambridge, Cambridge, UK; 471 Prostate Cancer Canada, Toronto, ON Canada; 472 University of Cambridge, Cambridge, UK; 473 Department of Laboratory Medicine, Translational Cancer Research, Lund University Cancer Center at Medicon Village, Lund University, Lund, Sweden; 474 Heidelberg University, Heidelberg, Germany; 475 New BIH Digital Health Center, Berlin Institute of Health (BIH) and Charité - Universitätsmedizin Berlin, Berlin, Germany; 476 CIBER Epidemiología y Salud Pública (CIBERESP), Madrid, Spain; 477 Research Group on Statistics, Econometrics and Health (GRECS), UdG, Barcelona, Spain; 478 Quantitative Genomics Laboratories (qGenomics), Barcelona, Spain; 479 Icelandic Cancer Registry, Icelandic Cancer Society, Reykjavik, Iceland; 480 State Key Laboratory of Cancer Biology, and Xijing Hospital of Digestive Diseases, Fourth Military Medical University, Shaanxi, China; 481 Department of Medicine (DIMED), Surgical Pathology Unit, University of Padua, Padua, Italy; 482 Rigshospitalet, Copenhagen, Denmark; 483 Center for Cancer Genomics, National Cancer Institute, National Institutes of Health, Bethesda, MD USA; 484 Department of Biochemistry and Molecular Medicine, University of Montreal, Montreal, QC Canada; 485 Australian Institute of Tropical Health and Medicine, James Cook University, Douglas, QLD Australia; 486 Department of Neuro-Oncology, Istituto Neurologico Besta, Milano, Italy; 487 Bioplatforms Australia, North Ryde, NSW Australia; 488 Department of Pathology (Research), University College London Cancer Institute, London, UK; 489 Department of Surgical Oncology, Princess Margaret Cancer Centre, Toronto, ON Canada; 490 Department of Medical Oncology, Josephine Nefkens Institute and Cancer Genomics Centre, Erasmus Medical Center, Rotterdam, CN The Netherlands; 491 The University of Queensland Thoracic Research Centre, The Prince Charles Hospital, Brisbane, QLD Australia; 492 CIBIO/InBIO - Research Center in Biodiversity and Genetic Resources, Universidade do Porto, Vairão, Portugal; 493 HCA Laboratories, London, UK; 494 University of Liverpool, Liverpool, UK; 495 The Azrieli Faculty of Medicine, Bar-Ilan University, Safed, Israel; 496 Department of Neurosurgery, University of Florida, Gainesville, FL USA; 497 Department of Pathology, Graduate School of Medicine, University of Tokyo, Tokyo, Japan; 498 University of Milano Bicocca, Monza, Italy; 499 BGI-Shenzhen, Shenzhen, China; 500 Department of Pathology, Oslo University Hospital Ulleval, Oslo, Norway; 501 Center for Biomedical Informatics, Harvard Medical School, Boston, MA USA; 502 Department Biochemistry and Molecular Biomedicine, University of Barcelona, Barcelona, Spain; 503 Office of Cancer Genomics, National Cancer Institute, National Institutes of Health, Bethesda, MD USA; 504 Cancer Epigenomics, German Cancer Research Center (DKFZ), Heidelberg, Germany; 505 Department of Cancer Biology, The University of Texas MD Anderson Cancer Center, Houston, TX USA; 506 Department of Surgical Oncology, The University of Texas MD Anderson Cancer Center, Houston, TX USA; 507 Department of Computer Science, Yale University, New Haven, CT USA; 508 Department of Molecular Biophysics and Biochemistry, Yale University, New Haven, CT USA; 509 Program in Computational Biology and Bioinformatics, Yale University, New Haven, CT USA; 510 Center for Cancer Research, Massachusetts General Hospital, Boston, MA USA; 511 Department of Pathology, Massachusetts General Hospital, Boston, MA USA; 512 Department of Pathology, Memorial Sloan Kettering Cancer Center, New York, NY USA; 513 Division of Gastroenterology and Hepatology, Mayo Clinic, Rochester, MN USA; 514 University of Sydney, Sydney, NSW Australia; 515 University of Oxford, Oxford, UK; 516 Department of Surgery, Academic Urology Group, University of Cambridge, Cambridge, UK; 517 Department of Medicine II, University of Würzburg, Wuerzburg, Germany; 518 Sylvester Comprehensive Cancer Center, University of Miami, Miami, FL USA; 519 Institut Hospital del Mar d’Investigacions Mèdiques (IMIM), Barcelona, Spain; 520 Genome Integrity and Structural Biology Laboratory, National Institute of Environmental Health Sciences (NIEHS), Durham, NC USA; 521 St. Thomas’s Hospital, London, UK; 522 Osaka International Cancer Center, Osaka, Japan; 523 Department of Pathology, Skåne University Hospital, Lund University, Lund, Sweden; 524 Department of Medical Oncology, Beatson West of Scotland Cancer Centre, Glasgow, UK; 525 National Human Genome Research Institute, National Institutes of Health, Bethesda, MD USA; 526 Centre for Cancer Research, Victorian Comprehensive Cancer Centre, University of Melbourne, Melbourne, VIC Australia; 527 Department of Medicine, Section of Hematology/Oncology, University of Chicago, Chicago, IL USA; 528 German Center for Infection Research (DZIF), Partner Site Hamburg-Borstel-Lübeck-Riems, Hamburg, Germany; 529 Bioinformatics Research Centre (BiRC), Aarhus University, Aarhus, Denmark; 530 Department of Biotechnology, Ministry of Science and Technology, Government of India, New Delhi, Delhi India; 531 National Cancer Centre Singapore, Singapore, Singapore; 532 Brandeis University, Waltham, MA USA; 533 Department of Urologic Sciences, University of British Columbia, Vancouver, BC Canada; 534 Department of Internal Medicine, Stanford University, Stanford, CA USA; 535 The University of Texas Health Science Center at Houston, Houston, TX USA; 536 Imperial College NHS Trust, Imperial College, London, INY UK; 537 Senckenberg Institute of Pathology, University of Frankfurt Medical School, Frankfurt, Germany; 538 Department of Medicine, Division of Biomedical Informatics, UC San Diego School of Medicine, San Diego, CA USA; 539 Center for Precision Health, School of Biomedical Informatics, The University of Texas Health Science Center, Houston, TX USA; 540 Oxford Nanopore Technologies, New York, NY USA; 541 Institute of Medical Science, University of Tokyo, Tokyo, Japan; 542 Howard Hughes Medical Institute, University of California Santa Cruz, Santa Cruz, CA USA; 543 Wakayama Medical University, Wakayama, Japan; 544 Department of Internal Medicine, Division of Medical Oncology, Lineberger Comprehensive Cancer Center, University of North Carolina at Chapel Hill, Chapel Hill, NC USA; 545 University of Tennessee Health Science Center for Cancer Research, Memphis, TN USA; 546 Department of Histopathology, Salford Royal NHS Foundation Trust, Salford, UK; 547 Faculty of Biology, Medicine and Health, University of Manchester, Manchester, UK; 548 BIOPIC, ICG and College of Life Sciences, Peking University, Beijing, China; 549 Peking-Tsinghua Center for Life Sciences, Peking University, Beijing, China; 550 Children’s Hospital of Philadelphia, Philadelphia, PA USA; 551 Department of Bioinformatics and Computational Biology and Department of Systems Biology, The University of Texas MD Anderson Cancer Center, Houston, TX USA; 552 Karolinska Institute, Stockholm, Sweden; 553 The Donnelly Centre, University of Toronto, Toronto, ON Canada; 554 Department of Medical Genetics, College of Medicine, Hallym University, Chuncheon, South Korea; 555 Department of Experimental and Health Sciences, Institute of Evolutionary Biology (UPF-CSIC), Universitat Pompeu Fabra, Barcelona, Spain; 556 Health Data Science Unit, University Clinics, Heidelberg, Germany; 557 Massachusetts General Hospital Center for Cancer Research, Charlestown, MA USA; 558 Hokkaido University, Sapporo, Japan; 559 Department of Pathology and Clinical Laboratory, National Cancer Center Hospital, Tokyo, Japan; 560 Department of Genetics, University of North Carolina at Chapel Hill, Chapel Hill, NC USA; 561 Computational Biology, Leibniz Institute on Aging - Fritz Lipmann Institute (FLI), Jena, Germany; 562 University of Melbourne Centre for Cancer Research, Melbourne, VIC Australia; 563 University of Nebraska Medical Center, Omaha, NE USA; 564 Syntekabio Inc, Daejeon, South Korea; 565 Department of Pathology, Academic Medical Center, Amsterdam, AZ The Netherlands; 566 China National GeneBank-Shenzhen, Shenzhen, China; 567 Division of Molecular Genetics, German Cancer Research Center (DKFZ), Heidelberg, Germany; 568 Division of Life Science and Applied Genomics Center, Hong Kong University of Science and Technology, Clear Water Bay, Hong Kong, China; 569 Icahn School of Medicine at Mount Sinai, New York, NY USA; 570 Geneplus-Shenzhen, Shenzhen, China; 571 School of Computer Science and Technology, Xi’an Jiaotong University, Xi’an, China; 572 AbbVie, North Chicago, IL USA; 573 Institute of Pathology, Charité – University Medicine Berlin, Berlin, Germany; 574 Centre for Translational and Applied Genomics, British Columbia Cancer Agency, Vancouver, BC Canada; 575 Edinburgh Royal Infirmary, Edinburgh, UK; 576 Berlin Institute for Medical Systems Biology, Max Delbrück Center for Molecular Medicine, Berlin, Germany; 577 Department of Pediatric Immunology, Hematology and Oncology, University Hospital, Heidelberg, Germany; 578 German Cancer Research Center (DKFZ), Heidelberg, Germany; 579 Heidelberg Institute for Stem Cell Technology and Experimental Medicine (HI-STEM), Heidelberg, Germany; 580 Institute for Computational Biomedicine, Weill Cornell Medical College, New York, NY USA; 581 New York Genome Center, New York, NY USA; 582 Department of Urology, James Buchanan Brady Urological Institute, Johns Hopkins University School of Medicine, Baltimore, MD USA; 583 Department of Preventive Medicine, Graduate School of Medicine, The University of Tokyo, Tokyo, Japan; 584 Department of Molecular and Cellular Biology, Baylor College of Medicine, Houston, TX USA; 585 Department of Pathology and Immunology, Baylor College of Medicine, Houston, TX USA; 586 Michael E. DeBakey Veterans Affairs Medical Center, Houston, TX USA; 587 Technical University of Denmark, Lyngby, Denmark; 588 Department of Pathology, College of Medicine, Hanyang University, Seoul, South Korea; 589 Academic Unit of Surgery, School of Medicine, College of Medical, Veterinary and Life Sciences, University of Glasgow, Glasgow Royal Infirmary, Glasgow, UK; 590 Department of Pathology, Asan Medical Center, College of Medicine, Ulsan University, Songpa-gu, Seoul South Korea; 591 Science Writer, Garrett Park, MD USA; 592 International Cancer Genome Consortium (ICGC)/ICGC Accelerating Research in Genomic Oncology (ARGO) Secretariat, Ontario Institute for Cancer Research, Toronto, ON Canada; 593 University of Ljubljana, Ljubljana, Slovenia; 594 Department of Public Health Sciences, University of Chicago, Chicago, IL USA; 595 Research Institute, NorthShore University HealthSystem, Evanston, IL USA; 596 Department for Biomedical Research, University of Bern, Bern, Switzerland; 597 Centre of Genomics and Policy, McGill University and Génome Québec Innovation Centre, Montreal, QC Canada; 598 Carolina Center for Genome Sciences, University of North Carolina at Chapel Hill, Chapel Hill, NC USA; 599 Hopp Children’s Cancer Center (KiTZ), Heidelberg, Germany; 600 Pediatric Glioma Research Group, German Cancer Research Center (DKFZ), Heidelberg, Germany; 601 Cancer Research UK, London, UK; 602 Indivumed GmbH, Hamburg, Germany; 603 Genome Integration Data Center, Syntekabio, Inc, Daejeon, South Korea; 604 University Hospital Zurich, Zurich, Switzerland; 605 Clinical Bioinformatics, Swiss Institute of Bioinformatics, Geneva, Switzerland; 606 Institute for Pathology and Molecular Pathology, University Hospital Zurich, Zurich, Switzerland; 607 Institute of Molecular Life Sciences, University of Zurich, Zurich, Switzerland; 608 MRC Human Genetics Unit, MRC IGMM, University of Edinburgh, Edinburgh, UK; 609 Women’s Cancer Program at the Samuel Oschin Comprehensive Cancer Institute, Cedars-Sinai Medical Center, Los Angeles, CA USA; 610 Department of Biology, Bioinformatics Group, Division of Molecular Biology, Faculty of Science, University of Zagreb, Zagreb, Croatia; 611 Department for Internal Medicine II, University Hospital Schleswig-Holstein, Kiel, Germany; 612 Genetics and Molecular Pathology, SA Pathology, Adelaide, SA Australia; 613 Department of Gastric Surgery, National Cancer Center Hospital, Tokyo, Japan; 614 Department of Bioinformatics, Division of Cancer Genomics, National Cancer Center Research Institute, Tokyo, Japan; 615 A.A. Kharkevich Institute of Information Transmission Problems, Moscow, Russia; 616 Oncology and Immunology, Dmitry Rogachev National Research Center of Pediatric Hematology, Moscow, Russia; 617 Skolkovo Institute of Science and Technology, Moscow, Russia; 618 Department of Surgery, The George Washington University, School of Medicine and Health Science, Washington, DC USA; 619 Endocrine Oncology Branch, Center for Cancer Research, National Cancer Institute, National Institutes of Health, Bethesda, MD USA; 620 Melanoma Institute Australia, Macquarie University, Sydney, NSW Australia; 621 MIT Computer Science and Artificial Intelligence Laboratory, Massachusetts Institute of Technology, Cambridge, MA USA; 622 Tissue Pathology and Diagnostic Oncology, Royal Prince Alfred Hospital, Sydney, NSW Australia; 623 Cholangiocarcinoma Screening and Care Program and Liver Fluke and Cholangiocarcinoma Research Centre, Faculty of Medicine, Khon Kaen University, Khon Kaen, Thailand; 624 Controlled Department and Institution, New York, NY USA; 625 Englander Institute for Precision Medicine, Weill Cornell Medicine, New York, NY USA; 626 National Cancer Center, Gyeonggi, South Korea; 627 Department of Biochemistry, College of Medicine, Ewha Womans University, Seoul, South Korea; 628 Health Sciences Department of Biomedical Informatics, University of California San Diego, La Jolla, CA USA; 629 Research Core Center, National Cancer Centre Korea, Goyang-si, South Korea; 630 Department of Health Sciences and Technology, Sungkyunkwan University School of Medicine, Seoul, South Korea; 631 Samsung Genome Institute, Seoul, South Korea; 632 Breast Oncology Program, Dana-Farber/Brigham and Women’s Cancer Center, Boston, MA USA; 633 Department of Surgery, Memorial Sloan Kettering Cancer Center, New York, NY USA; 634 Division of Breast Surgery, Brigham and Women’s Hospital, Boston, MA USA; 635 Integrative Bioinformatics Support Group, National Institute of Environmental Health Sciences (NIEHS), Durham, NC USA; 636 Department of Clinical Science, University of Bergen, Bergen, Norway; 637 Center For Medical Innovation, Seoul National University Hospital, Seoul, South Korea; 638 Department of Internal Medicine, Seoul National University Hospital, Seoul, South Korea; 639 Institute of Computer Science, Polish Academy of Sciences, Warsawa, Poland; 640 Functional and Structural Genomics, German Cancer Research Center (DKFZ), Heidelberg, Germany; 641 Laboratory of Translational Genomics, Division of Cancer Epidemiology and Genetics, National Cancer Institute, , National Institutes of Health, Bethesda, MD USA; 642 Institute for Medical Informatics Statistics and Epidemiology, University of Leipzig, Leipzig, Germany; 643 Morgan Welch Inflammatory Breast Cancer Research Program and Clinic, The University of Texas MD Anderson Cancer Center, Houston, TX USA; 644 Department of Hematology and Oncology, Georg-Augusts-University of Göttingen, Göttingen, Germany; 645 Institute of Cell Biology (Cancer Research), University of Duisburg-Essen, Essen, Germany; 646 King’s College London and Guy’s and St. Thomas’ NHS Foundation Trust, London, UK; 647 Center for Epigenetics, Van Andel Research Institute, Grand Rapids, MI USA; 648 The University of Queensland Centre for Clinical Research, Royal Brisbane and Women’s Hospital, Herston, QLD Australia; 649 Department of Pediatric Oncology and Hematology, University of Cologne, Cologne, Germany; 650 University of Düsseldorf, Düsseldorf, Germany; 651 Department of Pathology, Institut Jules Bordet, Brussels, Belgium; 652 Institute of Biomedicine, Sahlgrenska Academy at University of Gothenburg, Gothenburg, Sweden; 653 Children’s Medical Research Institute, Sydney, NSW Australia; 654 ILSbio, LLC Biobank, Chestertown, MD USA; 655 Division of Genetics and Genomics, Boston Children’s Hospital, Harvard Medical School, Boston, MA USA; 656 Institute for Bioengineering and Biopharmaceutical Research (IBBR), Hanyang University, Seoul, South Korea; 657 Department of Statistics, University of California Santa Cruz, Santa Cruz, CA USA; 658 National Genotyping Center, Institute of Biomedical Sciences, Academia Sinica, Taipei, Taiwan; 659 Department of Vertebrate Genomics/Otto Warburg Laboratory Gene Regulation and Systems Biology of Cancer, Max Planck Institute for Molecular Genetics, Berlin, Germany; 660 McGill University and Genome Quebec Innovation Centre, Montreal, QC Canada; 661 biobyte solutions GmbH, Heidelberg, Germany; 662 Gynecologic Oncology, NYU Laura and Isaac Perlmutter Cancer Center, New York University, New York, NY USA; 663 Division of Oncology, Stem Cell Biology Section, Washington University School of Medicine, St. Louis, MO USA; 664 Department of Systems Biology, The University of Texas MD Anderson Cancer Center, Houston, TX USA; 665 Harvard University, Cambridge, MA USA; 666 Urologic Oncology Branch, Center for Cancer Research, National Cancer Institute, National Institutes of Health, Bethesda, MD USA; 667 University of Oslo, Oslo, Norway; 668 University of Toronto, Toronto, ON Canada; 669 Peking University, Beijing, China; 670 School of Life Sciences, Peking University, Beijing, China; 671 Leidos Biomedical Research, Inc, McLean, VA USA; 672 Hematology, Hospital Clinic, Institut d’Investigacions Biomèdiques August Pi i Sunyer (IDIBAPS), University of Barcelona, Barcelona, Spain; 673 Second Military Medical University, Shanghai, China; 674 Chinese Cancer Genome Consortium, Shenzhen, China; 675 Department of Medical Oncology, Beijing Hospital, Beijing, China; 676 Laboratory of Molecular Oncology, Key Laboratory of Carcinogenesis and Translational Research (Ministry of Education), Peking University Cancer Hospital and Institute, Beijing, China; 677 School of Medicine/School of Mathematics and Statistics, University of St. Andrews, St, Andrews, Fife UK; 678 Institute for Systems Biology, Seattle, WA USA; 679 Department of Biochemistry and Molecular Biology, Faculty of Medicine, University Institute of Oncology-IUOPA, Oviedo, Spain; 680 Institut Bergonié, Bordeaux, France; 681 Cancer Unit, MRC University of Cambridge, Cambridge, UK; 682 Department of Pathology and Laboratory Medicine, Center for Personalized Medicine, Children’s Hospital Los Angeles, Los Angeles, CA USA; 683 John Curtin School of Medical Research, Canberra, ACT Australia; 684 MVZ Department of Oncology, PraxisClinic am Johannisplatz, Leipzig, Germany; 685 Department of Information Technology, Ghent University, Ghent, Belgium; 686 Department of Plant Biotechnology and Bioinformatics, Ghent University, Ghent, Belgium; 687 Institute for Genomic Medicine, Nationwide Children’s Hospital, Columbus, OH USA; 688 Computational Biology Program, School of Medicine, Oregon Health and Science University, Portland, OR USA; 689 Department of Surgery, Duke University, Durham, NC USA; 690 Institució Catalana de Recerca i Estudis Avançats (ICREA), Barcelona, Spain; 691 Institut Català de Paleontologia Miquel Crusafont, Universitat Autònoma de Barcelona, Barcelona, Spain; 692 University of Glasgow, Glasgow, UK; 693 Institut d’Investigacions Biomèdiques August Pi i Sunyer (IDIBAPS), Barcelona, Spain; 694 Division of Oncology, Washington University School of Medicine, St. Louis, MO USA; 695 Department of Surgery and Cancer, Imperial College, London, INY UK; 696 Applications Department, Oxford Nanopore Technologies, Oxford, UK; 697 Department of Obstetrics, Gynecology and Reproductive Services, University of California San Francisco, San Francisco, CA USA; 698 Department of Biochemistry and Molecular Medicine, University California at Davis, Sacramento, CA USA; 699 STTARR Innovation Facility, Princess Margaret Cancer Centre, Toronto, ON Canada; 700 Discipline of Surgery, Western Sydney University, Penrith, NSW Australia; 701 Yale School of Medicine, Yale University, New Haven, CT USA; 702 Department of Genetics, Lineberger Comprehensive Cancer Center, University of North Carolina at Chapel Hill, Chapel Hill, NC USA; 703 Departments of Neurology and Neurosurgery, Henry Ford Hospital, Detroit, MI USA; 704 Precision Oncology, OHSU Knight Cancer Institute, Oregon Health and Science University, Portland, OR USA; 705 Institute of Pathology, University Medical Center Hamburg-Eppendorf, Hamburg, Germany; 706 Department of Health Sciences, Faculty of Medical Sciences, Kyushu University, Fukuoka, Japan; 707 Heidelberg Academy of Sciences and Humanities, Heidelberg, Germany; 708 Department of Clinical Pathology, University of Melbourne, Melbourne, VIC, Australia; 709 Department of Pathology, Roswell Park Cancer Institute, Buffalo, NY USA; 710 Department of Computer Science, University of Helsinki, Helsinki, Finland; 711 Institute of Biotechnology, University of Helsinki, Helsinki, Finland; 712 Organismal and Evolutionary Biology Research Programme, University of Helsinki, Helsinki, Finland; 713 Department of Obstetrics and Gynecology, Division of Gynecologic Oncology, Washington University School of Medicine, St. Louis, MO USA; 714 Penrose St. Francis Health Services, Colorado Springs, CO USA; 715 Institute of Pathology, Ulm University and University Hospital of Ulm, Ulm, Germany; 716 National Cancer Center, Tokyo, Japan; 717 Genome Institute of Singapore, Singapore, Singapore; 718 32Program in Computational Biology and Bioinformatics, Yale University, New Haven, CT USA; 719 German Cancer Aid, Bonn, Germany; 720 Programme in Cancer and Stem Cell Biology, Centre for Computational Biology, Duke-NUS Medical School, Singapore, Singapore; 721 The Chinese University of Hong Kong, Shatin, NT, Hong Kong China; 722 Fourth Military Medical University, Shaanxi, China; 723 The University of Cambridge School of Clinical Medicine, Cambridge, UK; 724 St. Jude Children’s Research Hospital, Memphis, TN USA; 725 University Health Network, Princess Margaret Cancer Centre, Toronto, ON Canada; 726 Center for Biomolecular Science and Engineering, University of California Santa Cruz, Santa Cruz, CA USA; 727 Department of Medicine, University of Chicago, Chicago, IL USA; 728 Department of Neurology, Mayo Clinic, Rochester, MN USA; 729 Cambridge Oesophagogastric Centre, Cambridge University Hospitals NHS Foundation Trust, Cambridge, UK; 730 Department of Computer Science, Carleton College, Northfield, MN USA; 731 Institute of Cancer Sciences, College of Medical Veterinary and Life Sciences, University of Glasgow, Glasgow, UK; 732 Department of Epidemiology, University of Alabama at Birmingham, Birmingham, AL USA; 733 HudsonAlpha Institute for Biotechnology, Huntsville, AL USA; 734 O’Neal Comprehensive Cancer Center, University of Alabama at Birmingham, Birmingham, AL USA; 735 Department of Pathology, Keio University School of Medicine, Tokyo, Japan; 736 Department of Hepatobiliary and Pancreatic Oncology, National Cancer Center Hospital, Tokyo, Japan; 737 Sage Bionetworks, Seattle, WA USA; 738 Lymphoma Genomic Translational Research Laboratory, National Cancer Centre, Singapore, Singapore; 739 Department of Clinical Pathology, Robert-Bosch-Hospital, Stuttgart, Germany; 740 Department of Cell and Systems Biology, University of Toronto, Toronto, ON Canada; 741 Department of Biosciences and Nutrition, Karolinska Institutet, Stockholm, Sweden; 742 Center for Liver Cancer, Research Institute and Hospital, National Cancer Center, Gyeonggi, South Korea; 743 Division of Hematology-Oncology, Samsung Medical Center, Sungkyunkwan University School of Medicine, Seoul, South Korea; 744 Samsung Advanced Institute for Health Sciences and Technology, Sungkyunkwan University School of Medicine, Seoul, South Korea; 745 Cheonan Industry-Academic Collaboration Foundation, Sangmyung University, Cheonan, South Korea; 746 NYU Langone Medical Center, New York, NY USA; 747 Department of Hematology and Medical Oncology, Cleveland Clinic, Cleveland, OH USA; 748 Department of Radiation Oncology, University of California San Francisco, San Francisco, CA USA; 749 Department of Health Sciences Research, Mayo Clinic, Rochester, MN USA; 750 Helen F. Graham Cancer Center at Christiana Care Health Systems, Newark, DE USA; 751 Heidelberg University Hospital, Heidelberg, Germany; 752 CSRA Incorporated, Fairfax, VA USA; 753 Research Department of Pathology, University College London Cancer Institute, London, UK; 754 Department of Research Oncology, Guy’s Hospital, King’s Health Partners AHSC, King’s College London School of Medicine, London, UK; 755 Faculty of Medicine and Health Sciences, Macquarie University, Sydney, NSW Australia; 756 University Hospital of Minjoz, INSERM UMR 1098, Besançon, France; 757 Spanish National Cancer Research Centre, Madrid, Spain; 758 Center of Digestive Diseases and Liver Transplantation, Fundeni Clinical Institute, Bucharest, Romania; 759 Cureline, Inc, South San Francisco, CA USA; 760 St. Luke’s Cancer Centre, Royal Surrey County Hospital NHS Foundation Trust, Guildford, UK; 761 Cambridge Breast Unit, Addenbrooke’s Hospital, Cambridge University Hospital NHS Foundation Trust and NIHR Cambridge Biomedical Research Centre, Cambridge, UK; 762 East of Scotland Breast Service, Ninewells Hospital, Aberdeen, UK; 763 Department of Genetics, Microbiology and Statistics, University of Barcelona, IRSJD, IBUB, Barcelona, Spain; 764 Department of Obstetrics and Gynecology, Medical College of Wisconsin, Milwaukee, WI USA; 765 Hematology and Medical Oncology, Winship Cancer Institute of Emory University, Atlanta, GA USA; 766 Department of Computer Science, Princeton University, Princeton, NJ USA; 767 Vanderbilt Ingram Cancer Center, Vanderbilt University, Nashville, TN USA; 768 Ohio State University College of Medicine and Arthur G. James Comprehensive Cancer Center, Columbus, OH USA; 769 Department of Surgery, Yokohama City University Graduate School of Medicine, Kanagawa, Japan; 770 Division of Chromatin Networks, German Cancer Research Center (DKFZ) and BioQuant, Heidelberg, Germany; 771 Research Computing Center, University of North Carolina at Chapel Hill, Chapel Hill, NC USA; 772 School of Molecular Biosciences and Center for Reproductive Biology, Washington State University, Pullman, WA USA; 773 Finsen Laboratory and Biotech Research and Innovation Centre (BRIC), University of Copenhagen, Copenhagen, Denmark; 774 Department of Laboratory Medicine and Pathobiology, University of Toronto, Toronto, ON Canada; 775 Department of Pathology, Human Oncology and Pathogenesis Program, Memorial Sloan Kettering Cancer Center, New York, NY USA; 776 University Hospital Giessen, Pediatric Hematology and Oncology, Giessen, Germany; 777 Oncologie Sénologie, ICM Institut Régional du Cancer, Montpellier, France; 778 Institute of Clinical Molecular Biology, Christian-Albrechts-University, Kiel, Germany; 779 Institute of Pathology, University of Wuerzburg, Wuerzburg, Germany; 780 Department of Urology, North Bristol NHS Trust, Bristol, UK; 781 SingHealth, Duke-NUS Institute of Precision Medicine, National Heart Centre Singapore, Singapore, Singapore; 782 Department of Computer Science, University of Toronto, Toronto, ON Canada; 783 Bern Center for Precision Medicine, University Hospital of Bern, University of Bern, Bern, Switzerland; 784 Englander Institute for Precision Medicine, Weill Cornell Medicine and New York Presbyterian Hospital, New York, NY USA; 785 Meyer Cancer Center, Weill Cornell Medicine, New York, NY USA; 786 Pathology and Laboratory, Weill Cornell Medical College, New York, NY USA; 787 Vall d’Hebron Institute of Oncology: VHIO, Barcelona, Spain; 788 General and Hepatobiliary-Biliary Surgery, Pancreas Institute, University and Hospital Trust of Verona, Verona, Italy; 789 National Centre for Biological Sciences, Tata Institute of Fundamental Research, Bangalore, India; 790 Indiana University, Bloomington, IN USA; 791 Department of Pathology, GZA-ZNA Hospitals, Antwerp, Belgium; 792 Analytical Biological Services, Inc, Wilmington, DE USA; 793 Sydney Medical School, University of Sydney, Sydney, NSW Australia; 794 cBio Center, Dana-Farber Cancer Institute, Harvard Medical School, Boston, MA USA; 795 Department of Cell Biology, Harvard Medical School, Boston, MA USA; 796 Advanced Centre for Treatment Research and Education in Cancer, Tata Memorial Centre, Navi Mumbai, Maharashtra India; 797 School of Environmental and Life Sciences, Faculty of Science, The University of Newcastle, Ourimbah, NSW Australia; 798 Department of Dermatology, University Hospital of Essen, Essen, Germany; 799 Bioinformatics and Omics Data Analytics, German Cancer Research Center (DKFZ), Heidelberg, Germany; 800 Department of Urology, Charité Universitätsmedizin Berlin, Berlin, Germany; 801 Martini-Clinic, Prostate Cancer Center, University Medical Center Hamburg-Eppendorf, Hamburg, Germany; 802 Department of General Internal Medicine, University of Kiel, Kiel, Germany; 803 German Cancer Consortium (DKTK), Partner site Berlin, Berlin, Germany; 804 Cancer Research Institute, Beth Israel Deaconess Medical Center, Boston, MA USA; 805 University of Pittsburgh, Pittsburgh, PA USA; 806 Department of Ophthalmology and Ocular Genomics Institute, Massachusetts Eye and Ear, Harvard Medical School, Boston, MA USA; 807 Center for Psychiatric Genetics, NorthShore University HealthSystem, Evanston, IL USA; 808 Van Andel Research Institute, Grand Rapids, MI USA; 809 Laboratory of Molecular Medicine, Human Genome Center, Institute of Medical Science, University of Tokyo, Tokyo, Japan; 810 Japan Agency for Medical Research and Development, Tokyo, Japan; 811 Korea University, Seoul, South Korea; 812 Murtha Cancer Center, Walter Reed National Military Medical Center, Bethesda, MD USA; 813 Human Genetics, University of Kiel, Kiel, Germany; 814 Department of Oncologic Pathology, Dana-Farber Cancer Institute, Harvard Medical School, Boston, MA USA; 815 Oregon Health and Science University, Portland, OR USA; 816 Center for RNA Interference and Noncoding RNA, The University of Texas MD Anderson Cancer Center, Houston, TX USA; 817 Department of Experimental Therapeutics, The University of Texas MD Anderson Cancer Center, Houston, TX USA; 818 Department of Gynecologic Oncology and Reproductive Medicine, The University of Texas MD Anderson Cancer Center, Houston, TX USA; 819 University Hospitals Coventry and Warwickshire NHS Trust, Coventry, UK; 820 Department of Radiation Oncology, Radboud University Nijmegen Medical Centre, Nijmegen, GA The Netherlands; 821 Institute for Genomics and Systems Biology, University of Chicago, Chicago, IL USA; 822 Clinic for Hematology and Oncology, St.-Antonius-Hospital, Eschweiler, Germany; 823 Computational and Systems Biology Program, Memorial Sloan Kettering Cancer Center, New York, NY USA; 824 University of Iceland, Reykjavik, Iceland; 825 Division of Computational Genomics and Systems Genetics, German Cancer Research Center (DKFZ), Heidelberg, Germany; 826 Dundee Cancer Centre, Ninewells Hospital, Dundee, UK; 827 Department for Internal Medicine III, University of Ulm and University Hospital of Ulm, Ulm, Germany; 828 Institut Curie, INSERM Unit 830, Paris, France; 829 Department of Gastroenterology and Hepatology, Yokohama City University Graduate School of Medicine, Kanagawa, Japan; 830 Department of Laboratory Medicine, Radboud University Nijmegen Medical Centre, Nijmegen, GA The Netherlands; 831 Division of Cancer Genome Research, German Cancer Research Center (DKFZ), Heidelberg, Germany; 832 Department of General Surgery, Singapore General Hospital, Singapore, Singapore; 833 Cancer Science Institute of Singapore, National University of Singapore, Singapore, Singapore; 834 Department of Medical and Clinical Genetics, Genome-Scale Biology Research Program, University of Helsinki, Helsinki, Finland; 835 East Anglian Medical Genetics Service, Cambridge University Hospitals NHS Foundation Trust, Cambridge, UK; 836 Irving Institute for Cancer Dynamics, Columbia University, New York, NY USA; 837 Institute of Molecular and Cell Biology, Singapore, Singapore; 838 Laboratory of Cancer Epigenome, Division of Medical Science, National Cancer Centre Singapore, Singapore, Singapore; 839 Universite Lyon, INCa-Synergie, Centre Léon Bérard, Lyon, France; 840 Department of Urology, Mayo Clinic, Rochester, MN USA; 841 Royal National Orthopaedic Hospital - Stanmore, Stanmore, Middlesex UK; 842 Department of Biochemistry, Genetics and Immunology, University of Vigo, Vigo, Spain; 843 Giovanni Paolo II / I.R.C.C.S. Cancer Institute, Bari, BA Italy; 844 Neuroblastoma Genomics, German Cancer Research Center (DKFZ), Heidelberg, Germany; 845 Fondazione Policlinico Universitario Gemelli IRCCS, Rome, Italy, Rome, Italy; 846 University of Verona, Verona, Italy; 847 Centre National de Génotypage, CEA - Institute de Génomique, Evry, France; 848 CAPHRI Research School, Maastricht University, Maastricht, ER The Netherlands; 849 Department of Biopathology, Centre Léon Bérard, Lyon, France; 850 Université Claude Bernard Lyon 1, Villeurbanne, France; 851 Core Research for Evolutional Science and Technology (CREST), JST, Tokyo, Japan; 852 Department of Biological Sciences, Laboratory for Medical Science Mathematics, Graduate School of Science, University of Tokyo, Yokohama, Japan; 853 Department of Medical Science Mathematics, Medical Research Institute, Tokyo Medical and Dental University (TMDU), Tokyo, Japan; 854 Cancer Ageing and Somatic Mutation Programme, Wellcome Sanger Institute, Hinxton, UK; 855 University Hospitals Birmingham NHS Foundation Trust, Birmingham, UK; 856 Centre for Cancer Research and Cell Biology, Queen’s University, Belfast, UK; 857 Breast Medical Oncology, The University of Texas MD Anderson Cancer Center, Houston, TX USA; 858 Department of Surgery, Johns Hopkins University School of Medicine, Baltimore, MD USA; 859 Department of Oncology-Pathology, Science for Life Laboratory, Karolinska Institute, Stockholm, Sweden; 860 School of Cancer Sciences, Faculty of Medicine, University of Southampton, Southampton, UK; 861 Department of Gene Technology, Tallinn University of Technology, Tallinn, Estonia; 862 Genetics and Genome Biology Program, SickKids Research Institute, The Hospital for Sick Children, Toronto, ON Canada; 863 Departments of Neurosurgery and Hematology and Medical Oncology, Winship Cancer Institute and School of Medicine, Emory University, Atlanta, GA USA; 864 Department of Clinical and Molecular Medicine, Faculty of Medicine and Health Sciences, Norwegian University of Science and Technology, Trondheim, Norway; 865 Argmix Consulting, North Vancouver, BC Canada; 866 Department of Information Technology, Ghent University, Interuniversitair Micro-Electronica Centrum (IMEC), Ghent, Belgium; 867 Nuffield Department of Surgical Sciences, John Radcliffe Hospital, University of Oxford, Oxford, UK; 868 Institute of Mathematics and Computer Science, University of Latvia, Riga, LV Latvia; 869 Discipline of Pathology, Sydney Medical School, University of Sydney, Sydney, NSW Australia; 870 Department of Applied Mathematics and Theoretical Physics, Centre for Mathematical Sciences, University of Cambridge, Cambridge, UK; 871 Department of Epidemiology and Biostatistics, Memorial Sloan Kettering Cancer Center, New York, NY USA; 872 Department of Statistics, Columbia University, New York, NY USA; 873 Department of Immunology, Genetics and Pathology, Science for Life Laboratory, Uppsala University, Uppsala, Sweden; 874 School of Electronic and Information Engineering, Xi’an Jiaotong University, Xi’an, China; 875 Department of Histopathology, Cambridge University Hospitals NHS Foundation Trust, Cambridge, UK; 876 Oxford NIHR Biomedical Research Centre, University of Oxford, Oxford, UK; 877 Georgia Regents University Cancer Center, Augusta, GA USA; 878 Wythenshawe Hospital, Manchester, UK; 879 Department of Genetics, Washington University School of Medicine, St.Louis, MO USA; 880 Department of Biological Oceanography, Leibniz Institute of Baltic Sea Research, Rostock, Germany; 881 Wellcome Centre for Human Genetics, University of Oxford, Oxford, UK; 882 Department of Molecular and Human Genetics, Baylor College of Medicine, Houston, TX USA; 883 Thoracic Oncology Laboratory, Mayo Clinic, Rochester, MN USA; 884 Institute for Genomic Medicine, Nationwide Children’s Hospital, Columbus, OH USA; 885 Department of Obstetrics and Gynecology, Division of Gynecologic Oncology, Mayo Clinic, Rochester, MN USA; 886 International Institute for Molecular Oncology, Poznań, Poland; 887 Poznan University of Medical Sciences, Poznań, Poland; 888 Genomics and Proteomics Core Facility High Throughput Sequencing Unit, German Cancer Research Center (DKFZ), Heidelberg, Germany; 889 NCCS-VARI Translational Research Laboratory, National Cancer Centre Singapore, Singapore, Singapore; 890 Edison Family Center for Genome Sciences and Systems Biology, Washington University, St. Louis, MO USA; 891 MRC-University of Glasgow Centre for Virus Research, Glasgow, UK; 892 Department of Medical Informatics and Clinical Epidemiology, Division of Bioinformatics and Computational Biology, OHSU Knight Cancer Institute, Oregon Health and Science University, Portland, OR USA; 893 School of Electronic Information and Communications, Huazhong University of Science and Technology, Wuhan, China; 894 Department of Applied Mathematics and Statistics, Johns Hopkins University, Baltimore, MD USA; 895 Department of Cancer Genome Informatics, Graduate School of Medicine, Osaka University, Osaka, Japan; 896 Institute of Computer Science, Heidelberg University, Heidelberg, Germany; 897 School of Mathematics and Statistics, University of Sydney, Sydney, NSW Australia; 898 Ben May Department for Cancer Research, University of Chicago, Chicago, IL USA; 899 Department of Human Genetics, University of Chicago, Chicago, IL USA; 900 Tri-Institutional PhD Program in Computational Biology and Medicine, Weill Cornell Medicine, New York, NY USA; 901 The First Affiliated Hospital, Xi’an Jiaotong University, Xi’an, China; 902 Department of Medicine and Therapeutics, The Chinese University of Hong Kong, Shatin, NT, Hong Kong China; 903 Department of Biostatistics, The University of Texas MD Anderson Cancer Center, Houston, TX USA; 904 Duke-NUS Medical School, Singapore, Singapore; 905 Department of Surgery, Ruijin Hospital, Shanghai Jiaotong University School of Medicine, Shanghai, China; 906 School of Computing Science, University of Glasgow, Glasgow, UK; 907 Division of Orthopaedic Surgery, Oslo University Hospital, Oslo, Norway; 908 Eastern Clinical School, Monash University, Melbourne, VIC Australia; 909 Epworth HealthCare, Richmond, VIC Australia; 910 Department of Biostatistics and Computational Biology, Dana-Farber Cancer Institute and Harvard Medical School, Boston, MA USA; 911 Department of Biomedical Informatics, College of Medicine, The Ohio State University, Columbus, OH USA; 912 The Ohio State University Comprehensive Cancer Center (OSUCCC – James), Columbus, OH USA; 913 The University of Texas School of Biomedical Informatics (SBMI) at Houston, Houston, TX USA; 914 Department of Biostatistics, University of North Carolina at Chapel Hill, Chapel Hill, NC USA; 915 Department of Biochemistry and Molecular Genetics, Feinberg School of Medicine, Northwestern University, Chicago, IL USA; 916 Faculty of Medicine and Health, University of Sydney, Sydney, NSW Australia; 917 Department of Pathology, Erasmus Medical Center Rotterdam, Rotterdam, GD The Netherlands; 918 Division of Molecular Carcinogenesis, The Netherlands Cancer Institute, Amsterdam, CX The Netherlands; 919 Institute of Molecular Life Sciences and Swiss Institute of Bioinformatics, University of Zurich, Zurich, Switzerland

**Keywords:** Oncogenes, Medical genomics, Oncogenes

## Abstract

The discovery of driver mutations is one of the key motivations for cancer genome sequencing. *Here*, *as part of the ICGC/TCGA Pan-Cancer Analysis of Whole Genomes (PCAWG) Consortium*, which aggregated whole genome sequencing data from 2658 cancers across 38 tumour types, we describe DriverPower, a software package that uses mutational burden and functional impact evidence to identify driver mutations in coding and non-coding sites within cancer whole genomes. Using a total of 1373 genomic features derived from public sources, DriverPower’s background mutation model explains up to 93% of the regional variance in the mutation rate across multiple tumour types. By incorporating functional impact scores, we are able to further increase the accuracy of driver discovery. Testing across a collection of 2583 cancer genomes from the PCAWG project, DriverPower identifies 217 coding and 95 non-coding driver candidates. Comparing to six published methods used by the PCAWG Drivers and Functional Interpretation Working Group, DriverPower has the highest F1 score for both coding and non-coding driver discovery. This demonstrates that DriverPower is an effective framework for computational driver discovery.

## Abstract (web summary)

Analysis of cancer genome sequencing data has enabled the discovery of driver mutations. Here, as part of the ICGC/TCGA Pan-Cancer Analysis of Whole Genomes (PCAWG) Consortium the authors present DriverPower, a software package that identifies coding and non-coding driver mutations within cancer whole genomes via consideration of mutational burden and functional impact evidence.

## Introduction

Cancer drivers are somatic genetic alterations that confer selective advantages to tumour cells^1,2^. Identification of cancer drivers is a crucial yet challenging task in cancer genomics research^3,4^. There are multiple challenges. First, driver mutations generally account for only a small fraction of the somatic variations found in a typical tumour, the rest being innocent bystander ‘passenger' mutations^5^. Second, there is substantial intra- and inter-tumoural heterogeneity in most cancers^6^. Both across different tumour types and across different genomic regions within the same tumour, the background mutation rate (BMR) can vary over several orders of magnitude.

The advent of large-scale cancer whole-genome sequencing (WGS) data has made it possible to explore the role of driver events in non-coding regions. However, identifying non-coding driver events in WGS creates new challenges. First, although the functional impact of somatic mutations in the coding regions of genes is fairly straightforward to predict, much less is known about the effect of mutations on non-coding regions of the genome. Second, only ~1% of somatic mutations detected in PCAWG WGS data are exonic, adding substantially more mutations and regions to be tested and demanding more careful control of type I and type II errors than WGS. At present, only a limited number of non-coding drivers are known, the primary examples being the *TERT* promoter for multiple tumour types and the *TAL1* enhancer for T-cell acute lymphoblastic leukaemia^7,8^.

Most state-of-the-art methods identify drivers by detecting signals of positive selection either through mutational burden tests, which compare the rate of mutations observed in a region of the genome to what is expected from the BMR, or functional impact tests, which identify putative driver mutations based on a higher-than-expected rate of changes that are predicted to alter the function of genomic elements^3,6^. Mutational burden tests work best for calling frequently recurrent driver events and perform poorly when applied to rare driver events. In contrast, functional impact tests fail to find drivers in genomic elements that are poorly understood or annotated.

**Fig. 1:**
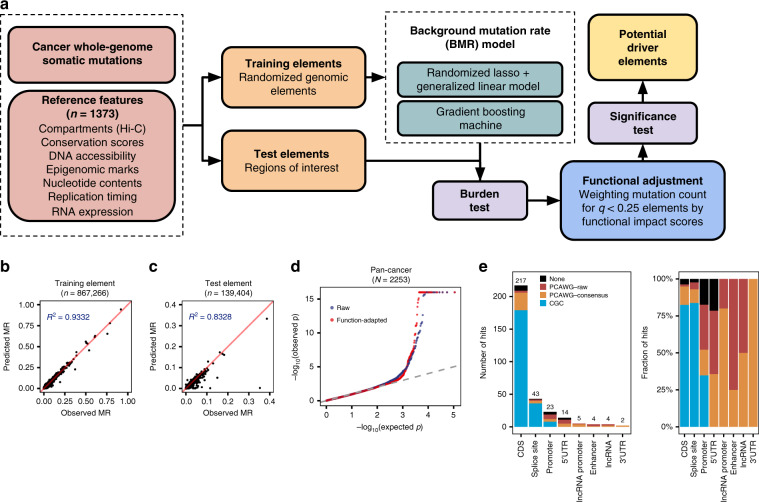
Summary of method and results. **a** DriverPower overview. **b**, **c** For the training and test element sets, comparison of the predicted (*Y* axis) and observed (*X* axis) mutation rate in the pan-cancer cohort. **d** The raw and function-adapted *p* value quantile-quantile (QQ)-plot for all test elements in the pan-cancer cohort. Function-adapted *p* values are *p* values with the incorporation of functional impact scores. **e** Number and fraction of non-coding driver candidates called by DriverPower contained within three reference driver sets (CGC, PCAWG-consensus or PCAWG-raw). For each element type, the number of candidates is also shown above the bar.

To maximise accuracy, we combined the two mutation significance testing methods to develop DriverPower (Fig. 1a), a framework for identification of coding and non-coding cancer drivers using mutational burden and functional impact scores. We first present the DriverPower method and describe the candidate driver mutations identified by applying the method to the ICGC/TCGA Pan-Cancer Analysis of Whole Genomes (PCAWG) data set. The PCAWG Consortium aggregated WGS data from 2658 cancers across 38 tumour types generated by the ICGC and TCGA projects. These sequencing data were re-analysed with standardised, high-accuracy pipelines to align to the human genome (reference build hs37d5) and identify germline variants and somatically acquired mutations, as described in ref. ^9^. Then we show that DriverPower outperforms several published methods for both coding and non-coding driver discovery and discuss some novel candidates identified by DriverPower.

## Results

### Features predictive of BMR

To evaluate DriverPower, we took WGS somatic variant data derived from 2583 high-quality donors from the PCAWG project^9^. After removing hypermutated samples, 2514 donors with 24,715,214 somatic single nucleotide variants (SNV) and small indels were used for driver element identification. We analysed these data both as a single pan-cancer data set, as well as a series of 29 tumour type-specific cohorts (Supplementary Data 1).

Among all tumour cohorts, we observed substantial variability in the observed mutation rate at the tissue, donor and locus levels (Supplementary Figs. 1 and 2). Accurate driver detection requires an accurate estimate of BMR across the tumour genome, taking into account the extensive variability among tumour types, donors and genomic regions. DriverPower tackles this issue by modelling the BMR using numerous genomic features that co-vary with the localised BMR. We collected 1373 features from three public data portals (Supplementary Data 2): the ROADMAP Epigenomics project, the ENCODE project and the UCSC genome browser^10–12^. These features covered seven main categories: conservation, DNA accessibility, epigenomic marks, nucleotide contents, replication timing, RNA expression and genome compartments. As expected, we found extensive multicollinearity among features. Most features (1368/1373) are significantly (Spearman’s rho test *q* < 0.1) correlated with pan-cancer genome-wide mutation rates (Supplementary Fig. 3).

### BMR model

We investigated two algorithms for modelling the BMR based on genomic features. The first algorithm was randomised lasso followed by binomial generalised linear model (GLM). The alternative algorithm was the gradient boosting machine (GBM), which is a non-linear and non-parametric tree ensemble algorithm^13^. To evaluate both BMR modelling algorithms, we made non-overlapped 1 megabase pair (Mbp) autosomal elements (*n* = 2521) as well as training genomic elements (*n* = 867,266) by sampling genomic coordinates randomly. The number of mutations per element was then predicted with fivefold cross validation (CV).

When evaluated using 1-Mbp autosomal elements, we found that both algorithms could accurately predict the BMR (Supplementary Figs. 4 and 5). In high mutational burden tumour cohorts, we observed essentially no difference between two algorithms, however GBM consistently outperformed GLM when applied to low mutational burden tumour cohorts (Supplementary Fig. 6). When evaluated on the training element set, in which the size of element varies from 100 bp to 1 Mbp, the prediction accuracy drops due to higher BMR variability, especially for low mutational burden tumour cohorts such as Myeloid-MPN and CNS-PiloAstro (Supplementary Fig. 6). However, for large cohort such as the pan-cancer set (*N* = 2253), ~ 93% of the mutation rate variance on the training set is explained by either model (Fig. 1b). The model still shows excellent performance when applied to the test element set, explaining 83% of the mutation rate variance on the pan-cancer cohort (Fig. 1c).

Both the randomised lasso algorithm and the GBM can be used to rank feature importance in different ways. Feature selection ranking from both methods confirmed that H3K9me3 (associated with heterochromatin), replication timing and H3K27ac (or its antagonistic histone mark H3K27me3) are the most important groups of predictors for BMR (Supplementary Fig. 7 and Supplementary Data 2)^14^. Consistent with previous results, we found that features from tumour cell lines with similar cell-of-origin to the primary tumour type are frequently selected^15^. For example, replication timing from liver cancer cell line HepG2 was selected as a feature for the BMR in hepatocellular carcinoma (Liver-HCC), whereas replication timing in MCF7 (breast cancer) and SK-N-SH (neuroblastoma) were selected for breast adenocarcinoma (Breast-AdenoCA) and glioblastoma (CNS-GBM), respectively (Supplementary Fig. 8).

### Functional adjustment

In most burden-based methods, mutations are equally weighted. However, not all mutations have the same functional consequences. To incorporate functional consequence information, DriverPower implements a posterior functional adjustment. The functional adjustment step up-weights mutations with high predicted functional impact. Althyough the DriverPower framework can potentially work with any functional scoring scheme, in the current implementation we measured the functional impact using four published scoring schemes: the CADD^16^, DANN^17^, EIGEN^18^ and LINSIGHT^19^ scores. Although different training data, assumptions and algorithms are used by different scores, we found those scores to be consistent at the element level (Supplementary Fig. 9). We used the average weight of all four scores in the remainder of the manuscript unless otherwise specified.

### Candidate driver event discovery

To evaluate the DriverPower algorithm, we first employed three simulated variant sets generated by the PCAWG Drivers and Functional Interpretation Working Group (PDFIWG) to examine type I and type II errors. We expected to identify no drivers as all three simulated data sets are reshuffles of observed mutations. In general, we observed no inflation or deflation in simulations and only eight significant hits (DriverPower *q* < 0.1) were identified in ~ 11 M statistical tests (Supplementary Fig. 10). We then used the observed PCAWG data set to discover drivers within multiple coding and non-coding element sets identified by the PDFIWG, spanning 3.7% (~ 113 Mbp) of the human genome.

We benchmarked our results against reference driver element sets and driver candidates called by six other published methods. Among the six methods, ExInAtor^20^, ncdDetect^21^ and LARVA^22^ use only mutational burden information; oncodriveFML^23^ uses only functional biases; whereas MutSig^24^ and ActiveDriverWGS^25^ model both mutational burden and functional consequence but not through functional impact scores. Three reference driver element sets were used: the COSMIC Cancer Gene Census (CGC)^26,27^, the PCAWG raw integrated driver candidates (PCAWG-raw) and the PCAWG consensus driver candidates (PCAWG-consensus). The CGC is a catalogue of driver genes for which mutations have been causally implicated in cancer and was used as the gold standard set (i.e., used in the calculation of precision and recall) for coding and splice site drivers. PCAWG-raw is an integration of driver elements called by 12 different driver detection methods on the same data we used here. PCAWG-consensus is a conservative set derived from the PCAWG-raw by applying multiple stringent filters to control the false discovery rate; in particular, the majority of non-coding candidates from lymphoid tumours and skin melanomas is excluded from this set because of hyper-mutational processes in these tumour types that create prominent mutational hotspots^28–30^. For the same reason our analysis of non-coding regions for tumour-specific and the pan-cancer cohorts excluded melanoma and lymphoma.

**Fig. 2:**
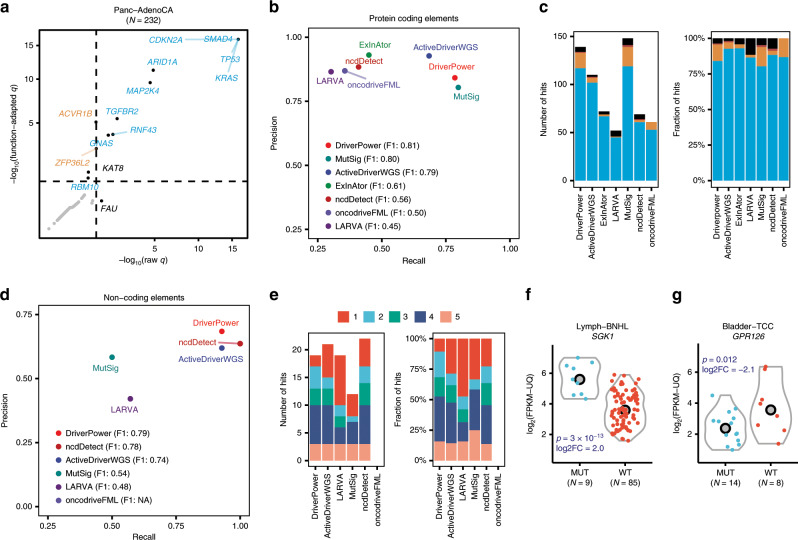
Benchmarking DriverPower driver discovery performance. **a** Comparison of CDS results with or without functional adjustment for Panc-AdenoCA. Dashed lines in **a** represent the *q* value = 0.1 threshold. Function-adapted *q* values are *q* values with the incorporation of functional impact scores. Only significant genes are labelled (colour legend is the same as Fig. 1e). **b**, **c** Benchmark results for coding genes compared with six other driver discovery methods. **d**, **e** Benchmark results for 3′-UTR, 5′-UTR, promoter and enhancer sets. **b**, d Show the precision and recall for each method according to results of 26 tumour cohorts (no melanoma and lymphoma). **c** Shows the number and fraction of coding driver candidates called by each method that are contained within reference gene sets. The coloured columns in **c** correspond to different reference driver sets (colour legend is the same as Fig. 1e). **e** Shows the number and fraction of non-coding driver candidates called by each method that are also called by others. The coloured columns in **e** correspond to the number of methods that agree on a driver candidate. **f** Differential expression analysis for the CDS and splice site of *SGK1* in Lymph-BNHL. **g** Differential expression analysis for the *GPR126* enhancer in Bladder-TCC. MUT indicates samples with mutated element and WT indicates samples without mutated element. Copy number corrected *p* values from the likelihood ratio test and the log2 fold changes (log2FC) are shown in blue.

Overall, we observed well-calibrated *p* values in DriverPower’s results with or without functional adjustment (Fig. 1d and Supplementary Fig. 10) and a high accuracy for both coding and non-coding driver discovery (Fig. 1e, Supplementary Data 3). For protein-coding regions (CDS), DriverPower found 217 significant (*q* < 0.1) driver candidates. As a gene (e.g., *TP53*) can be driver in multiple cohorts, the unique number of genes was 131. The precision of the algorithm’s driver calls was high. Among the driver genes called by DriverPower, 82.5% (179/217) of all genes were present within the CGC. For non-CGC genes, 27 and 3 genes were present within PCAWG-consensus and PCAWG-raw, respectively. Thus, only 3.7% (8/217) coding driver candidates called by DriverPower were not contained within any reference gene sets. As expected, incorporation of functional information increased both precision and recall in coding driver discovery (Fig. 2a and Supplementary Fig. 11). For example, in pancreatic ductal adenocarcinoma (Panc-AdenoCA; *N* = 232), the addition of functional adjustment to the algorithm resulted in a gain of three additional drivers (*ACVR1B*, *RBM10* and *ZFP36L2*) and the loss of one likely false-positive genes (*FAU*) (Fig. 2a). Without the use of functional information, the overall precision dropped to 74.6% (156/209) for CGC genes and 88.5% (185/209) for CGC/PCAWG genes. When compared with six other methods using the same 26 non-melanoma/lymphoma cohorts and CGC as the gold standard set, DriverPower (precision = 0.84; recall = 0.79) had the highest F1 score (0.81) (Fig. 2b, c). In our benchmark, sensitivity was a bottleneck for most methods (4/7 with recall < 0.5). When compared with the method with highest recall, the widely used coding driver caller MutSig (precision = 0.80; recall = 0.80), DriverPower identified an additional 21 genes present in CGC (23 for MutSig; Supplementary Fig. 12).

We next benchmarked DriverPower’s accuracy for non-coding driver events. For the prediction of driver events affecting the splice sites of coding genes, DriverPower called 47 significant (*q* < 0.1) candidates with 85.1% (40/47) within CGC. DriverPower (F1 = 0.91) also outperformed two recently published methods, ncdDetect (F1 = 0.65) and oncoDriverFML (F1 = 0.32), for splice site driver detection (Supplementary Fig. 13).

For the prediction of non-coding driver events in 3′-UTRs, 5′-UTRs, promoters and enhancers, DriverPower identified 19 candidates in non-melanoma/lymphoma tumour cohorts and 24 candidates in the pan-cancer cohort. Benchmarking results showed that DriverPower has the highest F1 score (0.79) among the six methods evaluated (Fig. 2d, e). Promoter and 5′-UTR driver candidates called by DriverPower were associated with a total of 17 unique genes. Of these, one gene (*TERT*) was in CGC, four genes (*WDR74*, *HES1*, *MTG2* and *PTDSS1*) were in PCAWG-consensus, and six other genes were in PCAWG-raw. DriverPower also called two 3′-UTR driver candidates in total, including *TOB1* in pan-cancer and *ALB* in Liver-HCC. Both candidates were present in the PCAWG-consensus. For enhancer regions, DriverPower identified two candidates: chr6:142,705,600-142,706,400 (linked to *GPR126*) and chr7:86,865,600-86,866,400 (linked to *TP53TG1*). Both enhancer elements were identified by PCAWG-raw; the *TP53TG1* enhancer was the only enhancer for non-melanoma/lymphoma tumours in the PCAWG-consensus set.

For long non-coding RNA (lncRNA) genes and their promoters, DriverPower found 9 candidates in total. Among them, 6 and 3 were contained within PCAWG-consensus and PCAWG-raw, respectively. These candidates targeted three unique lncRNAs: *RN7SK, RMRP* and *RPPH1*. The promoter of *RMRP* was significantly (*q* < 0.1) mutated in four cohorts (Breast-AdenoCA, Liver-HCC, Stomach-AdenoCA and pan-cancer) and has been nominated as a novel non-coding driver.

### DriverPower-exclusive driver candidates overview

A total of 11 coding and 17 unique non-coding candidates were exclusively identified by DriverPower (not present in either CGC or PCAWG-consensus; Supplementary Data 4). We sought to evaluate these exclusive driver candidates using literature evidence and correlative orthogonal data such as the effect of the variant on RNA-seq expression levels and the presence of somatic copy number alterations (SCNAs) and somatic structural variations (SVs) covering the same regions. On this basis, we found that many of the DriverPower-exclusive candidates are plausible cancer drivers.

Among protein-coding genes, DriverPower identified *EEF1A2* (eukaryotic translation elongation factor 1 alpha 2) in the oesophageal adenocarcinoma cohort (Eso-AdenoCA; 7/95 samples). All seven observed mutations were missense (Supplementary Fig. 14a). Although no RNA-seq data are available for Eso-AdenoCA samples, SCNA analysis indicated that *EEF1A2* is amplified in 69.5% (66/95) of Eso-AdenoCA samples (vs. 27.9% of non-Eso-AdenoCA samples; Supplementary Fig. 14b), suggesting a potential gain-of-function role in this cancer type. The amplification of *EEF1A2* (20q13.33) was also confirmed by the GISTIC2.0 (*q* = 0.0006). The same 1-Mbp locus detected by GISTIC2.0 was also amplified recurrently in other tumour types, including 73.1% of colorectal adenocarcinoma, 64.7% of stomach adenocarcinoma and 55.4% of ovarian adenocarcinoma. Supporting this hypothesis, previous studies have also demonstrated that *EEF1A2* is a putative oncogene in ovarian cancer and overexpressed in various tumour types^31–33^.

Another protein-coding gene exclusively identified by DriverPower was *MEF2B* in B-cell non-Hodgkin’s leukaemia (Lymph-BNHL; 8/105 samples). Among nine observed mutations, eight mutations were missense and one was a frameshift deletion (Supplementary Fig. 14c). RNA-seq data confirmed that mutated samples overexpressed *MEF2B* (copy number corrected *p* = 0.011; Supplementary Fig. 14d). *MEF2B* (Myocyte enhancer factor 2B) has been identified in multiple WES studies^34–36^, and a previous study has also shown that *MEF2B* mutations can dysregulate cell migration in non-Hodgkin lymphoma^37^.

One splice site candidate exclusively called by DriverPower is *SGK1* (serum/glucocorticoid regulated kinase 1) in Lymph-BNHL. The same gene was also significant in DriverPower’s CDS result for Lymph-BNHL (Supplementary Fig. 14e), resulting in a total of 13.3% (14/105) Lymph-BNHL samples being affected by non-synonymous or splice site mutations in *SGK1*. *SGK1* is present in PCAWG-raw but was filtered out owing to the large number of AID-related variants in this tumour cohort. However, differential expression analysis indicated that *SGK1* is significantly overexpressed in mutated Lymph-BNHL samples relative to non-mutated samples (copy number corrected *p* = 3e-13 from likelihood ratio test; Fig. 2f). *SGK1* encodes a serine/threonine protein kinase that has an important role in cellular stress response and its CDS has been nominated as a driver in earlier WES studies^35,36^. Another study has also demonstrated that the administration of an *SGK1* inhibitor induces apoptosis in lymphoma cell lines^38^. Together these data support a potential driver role for *SGK1* in Lymph-BNHL.

The *GPR126* (adhesion G protein-coupled receptor G6) enhancer candidate was filtered out from the PCAWG-raw set because of mutations in palindrome loops, which makes it unclear whether mutations in the *GPR126* enhancer are caused by mutational mechanism associated with palindrome loops or positive selection. We found that the *GPR126* enhancer is recurrently mutated in transitional cell carcinoma of the bladder (Bladder-TCC; 14/23 samples) and breast adenocarcinoma (Breast-AdenoCA; 8/195) (Supplementary Fig. 14f). *GPR126* is among the MammaPrint 70 gene panel used to predict the risk of breast cancer metastasis^39,40^. A study also shows that knockdown of *GPR126* can inhibit the hypoxia-induced angiogenesis in model organisms^41^. Differential expression analysis demonstrated that the *GPR126* is significantly downregulated in Bladder-TCC samples with enhancer mutations (copy number corrected *p* = 0.012 from likelihood ratio test; Fig. 2g) relative to those carrying the wild-type enhancer, suggesting a functional role for these mutations.

Several somatically altered histone genes have been implicated in human cancer, such as *H3F3A* (identified as a pan-cancer driver in this study), *H3F3B* and *HIST1H3B*^42–44^. DriverPower identified four histone genes as driver candidates in the pan-cancer cohort, two of which were absent from CGC or PCAWG-consensus: the 5′-UTR of *HIST1H2AC* and *HIST1H2BD*. Previous studies have shown that the protein levels of the replication-dependent histone H2A variant *HIST1H2AC* (encoding histone H2A type 1-C) is decreased in chronic lymphocytic leukaemia patients and bladder cancer cells^45,46^, and the siRNA knockdown of *HIST1H2AC* increases cell proliferation and promote oncogenesis^46^.

Several other driver candidates exclusively called by DriverPower are associated with genes that may have a role in cancer. The highly expressed liver-specific gene *ALB* (albumin) is significant (DriverPower *q* < 0.1) for somatic mutations affecting its CDS, splice site, 3′-UTR and promoter in Liver-HCC; the splice site and promoter (under CADD scores) were discovered by DriverPower exclusively. Correlative evidence from gene expression and copy number alterations suggested that loss-of-function mutations in *ALB* are subject to positive selection in Liver-HCC as described elsewhere^47^. The CDS of *KAT8* (lysine acetyltransferase 8) was called by DriverPower in Panc-AdenoCA with 100% (5/5) missense mutations. As a histone modifier, *KAT8* has been shown to physically interact with *MLL1* and regulate known cancer drivers *ATM* and *TP53*^48–51^. Previous studies have also shown that *KAT8* is downregulated in gastric cancer^52^ and *KAT8* can suppress tumour progression by inhibiting epithelial-to-mesenchymal transition^53^. The 5′-UTR and promoter of *SRSF9* (serine and arginine rich splicing factor 9) was significant in DriverPower’s results for pan-cancer and not present in any reference driver sets. The protein encoded by *SRSF9* is part of the spliceosome; a previous study indicates that the proto-oncogene *SRSF9* is overexpressed in multiple tumours and that this overexpression can cause the accumulation of β-Catenin^54^. The same study also showed that the depletion of *SRSF9* proteins could inhibit colon cancer cell proliferation.

In summary, 4/11 coding and 4/17 unique non-coding driver candidates exclusively called by DriverPower had some form of support from the literature or orthogonal evidence. If we assume that all the exclusive candidates that lack such evidence are false positives, then this puts an estimate of DriverPower’s false discovery rate across the PCAWG data set at 3.2% (7/217) for coding and 16.8% (16/95) for non-coding regions. However, this assumption is probably invalid as most of these lack-of-evidence candidates are also identified by other methods and present in PCAWG-raw. We acknowledge that lack-of-evidence candidates may contain false-positive calls, but they may also contain previously unknown drivers. For example, the 5′-UTR of *TBC1D12* in Breast-AdenoCA, which has been filtered out from the PCAWG-raw owing to possible hypermutability, is called by all but one driver discovery methods and is reported as a putative cancer driver in previous studies because of two recurrent mutations in the Kozak consensus sequence involving in the initiation of protein translation^23,55^. Moreover, according to another recent study, the same *TBC1D12* candidate is still statistically significant in breast cancer even after removing hypermutations, but whether these mutations can alter protein translation in cancer is still undetermined^24^. Some lack-of-evidence candidates may also fit the mini-driver model of cancer evolution^56^. Unlike classical drivers, mini-drivers can only weakly promote and are not essential for tumour progression, hence present at a lower frequency in cancer cohorts. Further investigation is required to determine the role of lack-of-evidence candidates in cancer.

### DriverPower applied to WGS

To demonstrate the robustness of DriverPower, we applied DriverPower to two public whole-exome sequencing (WES) data sets (Supplementary Fig. 15). Both WES data sets are processed differently than the PCAWG data and contain samples not included in the PCAWG study. For liver cancer, using models trained for Liver-HCC (*N* = 314), DriverPower identified 14 coding drivers from 364 TCGA-LIHC samples (53 shared with Liver-HCC). All but one driver candidates were present within the CGC or PCAWG-consensus. For pancreatic adenocarcinoma, using models trained for Panc-AdenoCA (*N* = 232), DriverPower identified six coding drivers from 180 TCGA-PAAD samples (no shared samples with the PCAWG study) and all corresponded to known driver genes.

## Discussion

Computational driver discovery is essential to distinguish driver from passenger mutations in the coding and non-coding regions of whole cancer genomes. Here we report DriverPower, a new framework for accurately identifying both types of driver mutation by combining mutational burden and functional impact information. The method takes advantage of the large somatic mutation sets produced by WGS technology to build an accurate global BMR model from more than a thousand genomic features. This contrasts with methods that build a local BMR model using selected or flanking regions. One advantage of this is that the method is not biased towards coding regions, but uses the same model for coding and non-coding cancer driver discovery. Another advantage is the method’s high degree of modularity. DriverPower can potentially work with any types of genomic element (contiguous or disjoint, coding or non-coding, proximate or distal to genes), any regression algorithms for modelling BMR and any functional impact score scheme. Although DriverPower is designed for WGS projects, it performs robustly in WES strategies as well.

In comparison with the other driver discovery methods evaluated by the PCAWG Drivers and Functional Interpretation Working Group, DriverPower provides the best balance of precision and recall, although is not always the top-ranked method when either metric is considered independently (Fig. 2b, d). As discussed in Supplementary Note 1, DriverPower is parameterised to allow for adjustment of the precision-recall trade-off; in this study, we selected conservative parameters that prioritise precision over recall especially for non-coding regions (Supplementary Fig. 16).

There are several ways in which the accuracy of DriverPower could be improved. One approach to improve recall is to take into the account the potential presence of negative (purifying) selection in the functional regions selected for testing. When the BMR model is trained, we use random genomic elements that are predominantly under neutral selection. However, the functional elements selected for testing are more likely to be under positive and/or negative selection^57^. The observed mutation rate reflects the balance between positive and negative selection, and negative selection at one site in the element will diminish the signal of positive selection at other sites, reducing the sensitivity of the method as a whole. To our knowledge, no driver discovery tool currently models the effect of negatively selected sites; future work aims to take this mechanism into account.

The precision of the method can also be improved. False-positive driver calls may be caused by technical errors such as variant-calling artefacts that artificially increase the local mutation rate, or by biological processes that are not captured by the BMR model such as regional differences in activation-induced cytidine deaminase (AID) activity. These can potentially be mitigated by incorporating into the BMR model additional features relevant to the technical and biological processes. For example, incorporating read-level coverage, mapping and bias scores into the BMR could help correct for regions prone to variant-calling artefacts, whereas features like the number of palindrome loops and the fraction of mutations caused by AID per element could be used to adjust for locally-acting hypermutation processes.

When applied to the PCAWG data set, DriverPower called nearly twice as many coding driver events as non-coding ones, a ratio also observed by the PCAWG driver study, a ratio also observed by the PCAWG driver study^47^. Although this unbalanced ratio may reflect cancer biology, there is also the possibility that it reflects, at least in part, the technical challenge of sequencing and interpreting non-coding regions. Potential artefacts include systematic undercalling of somatic variants in non-coding regions^24^, a problem that could be rectified by deeper coverage. For example, it is estimated that ~ 216 mutations in the *TERT* promoter are likely to be missed in the PCAWG data set owing to low detection sensitivity^47^. Another technical issue is raised by the fact that several non-coding candidates are only significant in the pan-cancer cohort, suggesting that the data set is statistically underpowered. In fact, although we studied 2583 genomes here, many tumour types have a sample size fewer than 30. To overcome this issue, we could either sequence more genomes or reduce the size of the set of test elements by narrowing it to functional motifs or conserved bases^58^. Moreover, only ~ 3.7% of the genome has been studied here. There may be more non-coding drivers in other types of regulatory elements, which demands more complete annotations for the non-coding part of the human genome. At last, functional impact score schemes are currently biased toward coding mutations; therefore, improved functional scoring schemes will also help us identify more functionally relevant non-coding cancer drivers in the future.

A comprehensive catalogue of coding and non-coding cancer drivers will accelerate the clinical translation of cancer genomic study to precision medicine. As more cancer genomes and more cancer types are sequenced, a general and accurate framework for computational driver discovery like DriverPower will become increasingly useful.

## Methods

### Ethical review

Sequencing of human subjects tissue was performed by ICGC and TCGA consortium members under a series of locally approved Institutional Review Board (IRB) protocols as described in Hudson et al.^59^. Informed consent was obtained from all human participants. Ethical review of the current data analysis project was granted by the University of Toronto Research Ethics Board (REB) under protocol #30278, ‘Pan-cancer Analysis of Whole Genomes: PCAWG'.

### Generation of cancer whole-genome somatic mutations

All DNA somatic SNVs and indels for 2583 donors were obtained from the PCAWG project (somatic variant callset released October 2016)^9^. For our analysis, donors with hypermutated signatures were excluded (*n* = 69, defined as > 30 mutations per Mb). Otherwise, we used the same type-specific (*n* = 29) and pan-cancer (all tumour samples except Skin-Melanoma, Lymph-NOS, Lymph-CLL and Lymph-BNHL) sample cohorts as the PCAWG Drivers and Functional Interpretation Working Group (PDFIWG; Supplementary Data 1)^47^.

### Generation of simulated somatic mutations

We used three simulated data sets (Broad, DKFZ and Sanger simulations) from the PDFIWG (described in detail at Rheinbay et al.^47^). These simulations were made to capture the variation of BMR and remove the signal of positive selection through permutations of observed somatic mutations.

### Generation of test and training genomic elements

We define a genomic element as the collection of genome coordinates that defines one specific functional region of interest. For example, the CDS element of *TP53* is the combination of all protein-coding regions in *TP53*.

We used eight test element sets in our analysis, including the CDS (*n* = 20,185), splice site (*n* = 18,729), 5′-UTR (*n* = 19,369), 3′-UTR (*n* = 19,188), promoter (*n* = 20,164), enhancer (*n* = 30,816), lncRNA (*n* = 5,580) and lncRNA promoter (*n* = 5373). All test element sets were obtained from the PCAWG project. GENCODE v19 was used as the reference gene model when building those sets^60^. Non-coding RNA annotations were collected from multiple sources as described.

We constructed genomic element training sets by randomly sampling genome coordinates from hg19, the build used for PCAWG. The length of each training element was sampled from the length distribution of test elements and multiplied by a factor of 3. Training elements overlapping test elements were removed. In total, 867,266 training elements were created and ~ 54% (1,545,491,997 bp) of the genome was covered.

### Collection and generation of features

We collected 1373 features in total. Details including data sources can be found at Supplementary Data 2. Nucleotide content features were calculated as the fraction of 2-mers and 3-mers in each genomic element. The number of 2-mers and 3-mers was counted directly from genome sequences (hg19). For raw features in bigwig format (typically genome-wide signals), we calculated the average signal strength of covered bases in each element using the bigWigAverageOverBed (v2) utility from the UCSC genome browser^61^. For raw features in BED format (typically narrow peaks of ChIP-seq data), we calculated the percentage of bases intersecting BED for each element with the BEDTools (v2.24.0)^62^. All missing values in features were filled with 0.

### The DriverPower outline

The main steps of the DriverPower (v1.0.0) framework are summarised below. Details of each step are described in following sections. The difference between v1.0.0 and the version used in the PDFIWG data analysis freeze (April 2017) is discussed in Supplementary Note 2 and Supplementary Fig. 17.

The first step of DriverPower is to scale features and/or filter out excluded regions. The second step is to build the BMR model using the GBM, or randomised lasso followed by binomial GLMs. The purpose of the BMR model is to estimate the expected number of mutations (*y*^) for any genomic element. Namely, we want to obtain $y_i^ \wedge = E\left( {y_i|X_i,L_i} \right)$ where *X*_*i*_ and *L*_*i*_ are the feature vector and length for the element *i*. The third step is to conduct burden test with observed (*y*) and predicted (*y*^) mutation counts, and perform multiple testing correction. The fourth step is to adjust observed mutation counts (*y*) based on functional impact scores for nearly significant elements (*q* < 0.25). The last step is to re-assess the significance for nearly significant elements with functional adjusted mutation counts followed by multiple testing correction.

### Scaling of features

Features were scaled with RobustScaler from scikit-learn (version 0.18)^63^. Feature scaling was only conducted for randomised lasso and GLMs.

### Definition of excluded regions

In this study, all bases in the excluded regions were removed before any analysis. The excluded region consists of three sets: (1) all *N* bases and gaps in the hg19 genome (fetched from the UCSC table browser^12^); (2) ENCODE consensus excludable regions (the DAC Blacklisted Regions track and the Duke Excluded Regions track from the UCSC genome browser)^64^; (3) PCAWG low mappability regions (data retrieved from the PCAWG variant group). PCAWG low mappability regions are defined as regions callable in fewer than 556/1111 (~ 50%) tumour-normal pairs. For each tumour-normal pair, a base is callable if there are more than 14/8 high quality reads in tumour/normal WGS. In total, 2,806,377,226 bp, or 96.41% of the genome are defined as callable.

### Feature selection with randomised lasso

To select features, we randomly sub-sampled 10% of the training set 500 times. Then for the *k*-th subset with size *N*_*k*_, the following model was fitted^65^:

$$w^ \wedge _k = arg\mathop {{min}}\limits_w \left( {\frac{1}{{N_k}}\left\| {logit\left( {\frac{{y + 1/2}}{{N \cdot L}}} \right) - Xw} \right\|_2^2 + \alpha \mathop {\sum }\limits_{i = 1}^{1373} \frac{{|w_i|}}{{b_i}}} \right)$$

where *N* is the total number of donors in the data set, *X* is the feature matrix, *w* is the weight vector, *α* is the regularisation parameter, and *b*_*i*_ is the scaling factor. The regularisation parameter *α* was determined by a fivefold CV lasso with 33% of the training data. For the *k*-th sub-sampling, the ith feature was selected if $w^ \wedge _{ki} \ge 0.001$. The final feature importance score was calculated as the fraction of times that a feature was selected. Only features with score > 0.5 were used in the GLM BMR model.

### Prediction of the BMR with GLM

When using the GLM, we modelled the observed mutations in each genomic element with a binomial distribution, that is

$$y \sim B(n,p)\,with\,n = N \cdot L\,and\,p = y^ \wedge /(N \cdot L)$$

where *y* is the observed mutation count and *y*^ is the estimated mutation count. We used the binomial GLM to obtain *y*^ with the logit link function, that is

$$\frac{{\hat y}}{{N \cdot L}} = E\left( {\frac{y}{{N \cdot L}}|X^{select}} \right) = logit^{ - 1}(X^{select}\beta )$$

where *X*^*select*^ is the selected feature matrix and *β* is the regression coefficient vector.

### Prediction of the BMR with GBM

We trained the GBM with XGBoost^66^. All features were used in model training. The negative Poisson log-likelihood was chosen as the objective function and $ln(N \cdot L)$ of elements were used as offset (i.e., base_margin in XGBoost). Other non-default parameters used in DriverPower were as follows: eta = 0.05, max_depth = 8, subsample = 0.6, max_delta_step = 1.2, early_stop_rounds = 5 and nrounds = 5000. The feature importance for GBM is measured by the improvement in accuracy brought by a feature across all trees. XGBoost returns feature importance that sums up to 1 for all features. We also normalised the importance to a [0, 1] scale (i.e., importance relative to the most important feature).

### Evaluation of two BMR models

We evaluated both models with 1 Mb autosome bins (*n* = 2521) and training genomic elements (*n* = 867,266) defined above. The 1 Mb elements have been used in many studies^14,15,67^. For both elements, we obtain the predicted mutation rate by fivefold CV. For 1 Mb elements, we used fourfold data for model training and onefold data for model evaluation. For training elements, we use 1-fold data to train the model and the rest to evaluate. As per previous work, we used *R*^2^ score and Pearson’s *r* as evaluation metrics^15^. Standard error of the mean (SEM) for *R*^2^ and *r* was calculated from fivefold CV.

### Calculation of element functional impact scores

Four different functional scores were used in this analysis^16–19^. For CDS, CADD (SNVs and indels, v1.3), DANN (SNVs) and EIGEN (SNVs) scores were used. CADD indel scores were generated with the CADD web interface for all observed indels in the PCAWG data set. For splice site, CADD and DANN scores were used. For non-coding elements, the CADD, DANN and LINSIGHT (SNVs and indels) score were used. Then the following steps were used to calculate the functional impact score per genomic element. First, raw scores were retrieved for all observed mutations in the data set. Second, all raw scores were converted to phred-like scores by $- 10log_{10}(rank/N_m)$, where *N*_*m*_ is the number of observed mutations having scores. Third, for each genomic element, its functional score *S* was calculated as:

$$S = \frac{1}{N}\mathop {\sum }\limits_{i = 1}^N \underline {s_i}$$

where *N* is the number of donors and *s*_*i*_ is the average functional impact score for the *i*th donor.

### Adjustment of the mutation count

To compensate for the unbalanced number of mutations among samples, instead of using the mutation count per element directly we used the geometric mean of mutation count and sample count. That is, we use the balanced count $y^b = \sqrt {y \cdot n_d}$ instead of *y* directly for significance test, where *n*_*d*_ is the number of mutated donors. Based on the motivation that not all mutations should be weighted the same, the balanced mutation count *y*^*b*^ was then adjusted for nearly significant elements (raw *q* value < 0.25) by a functional weight *w*, that is $y^f = w \cdot y^b$, where *y*^*f*^ is the functionally adjusted mutation count. For the element *j*, the functional weight *w*_*j*_ was calculated based on its functional score *S*_*j*_ and a threshold score *S*_*T*_:

$$w_j = \frac{{S_j}}{{S_T}} = \frac{{S_j}}{{ - 10log_{10}F}}$$

The threshold score *S*_*T*_ is controlled by a single parameter *F* between 0 and 1, and can be interpreted as the fraction of functionally relevant variants among all observed variants. Parameter tuning of *F* can be found at Supplementary Note 1.

### Assessment of the element significance

For each element, we calculated $P(y^b \ge y^ \wedge )$ as the raw *p* value and $P(y^f \ge y^ \wedge )$ as the function-adapted *p* value. As overdispersion has been documented in burden-based methods and can affect the driver discovery accuracy^22^, here we performed a regression-based overdispersion test for each tumour cohort using the training set^68^. Based on the result of the overdispersion test, we calculated the raw and function-adpated *p* values by following a binomial distribution or a negative binomial distribution:

$$y^b\,or\,y^f \sim \left\{ {NB(y^ \wedge ,s \cdot \theta ),if\,p \le 0.01\,B\left( {N \cdot L,\frac{{y^ \wedge }}{{N \cdot L}}} \right)} \right.$$

otherwise, where *p* and *θ* are the *p* value and dispersion parameter estimated from the overdispersion test, and *s* is the scaling factor for *θ* used to accommodate the discrepancy between test and training set in terms of the dispersion level. We used *s* = 3 for lymphomas and *s* = 1 otherwise in this analysis.

### Multiple testing correction

In all cases, *q* values were generated by the Benjamini–Hochberg procedure^69^. We chose *q* < 0.1 as the significant level and *q* < 0.25 as the nearly significant level. For each element set, multiple testing correction was performed for each tumour cohort (cohort *q* value) and across all tumour cohorts (global *q* value). Cohort *q* values were used in functional adjustment and global *q* values were used to define the final driver list.

### Generation of reference cancer drivers

Reference cancer drivers were used to benchmark the performance of DriverPower. Three reference sets were used: (1) the COSMIC CGC (v82, *n* = 567); (2) the PCAWG consensus driver candidates (PCAWG-consensus; *n* = 157 for coding and *n* = 26 for non-coding); (3) the PCAWG raw integrated driver candidates (PCAWG-raw; *n* = 193 for coding and *n* = 79 for non-coding). PCAWG-consensus (*q* value post-filtering < 0.1) is a set of highly confident non-coding drivers and subjected to multiple stringent filters as described. PCAWG-raw (*q* value pre-filtering < 0.1) is a superset of PCAWG-consensus and includes non-coding drivers that were not subjected to the filtering process. PCAWG-raw driver candidates that are mutated in fewer than three samples were removed in this analysis. For promoter and 5′-UTR candidates in the PCAWG consensus drivers, we reversed the filtering for overlapping elements (i.e., one element is selected over the overlapping element based on prior knowledge). For example, we kept both the promoter and the overlapping 5′-UTR of *WDR74* in this analysis; in the PCAWG consensus set, the *WDR74* promoter is preferentially selected over its 5′-UTR.

### Benchmarking of DriverPower

We compared coding and non-coding driver candidates called by DriverPower to driver candidates called by six other published driver detection tools (ActiveDriverWGS^25^, ExInAtor^20^, LARVA^22^, MutSig^24^, ncdDetect^21^ and oncodriveFML^23^). Driver calls for 26 single tumour cohorts (no Skin-Melanoma, Lymph-CLL and Lymph-BNHL) were retrieved from the PCAWG driver group. For each method, we removed driver candidates that are mutated in fewer than three samples. We used precision (TP/(TP + FP)), recall (TP/(TP + FN)) and F1 score (2*Precision*Recall/(Precision + Recall)) as performance metrics.

For CDS, we used the CGC gene set as the gold standard. For each method, true positive genes were defined as genes presented in the gold standard set and the precision was then calculated as the fraction of true positive genes among all called genes. For recall, since we cannot accurately know the expected set of driver genes that should be called for each tumour cohort in the data set, a lower-bound approximation was used instead. The lower-bound approximation was estimated by taking the union of all true positive genes identified by each method and the recall was then calculated as the fraction of true positive genes called among the lower-bound approximation.

For gene splice sites, the same gold standard gene set and benchmark method as CDS were used. Owing to data availability, the comparison was only performed for ncdDetect, oncodriveFML and DriverPower.

For promoters, enhancers, 3′-UTRs and 5′-UTRs, because the number of non-coding driver candidates is small, four element sets were benchmarked together. No data for ExInAtor is available for this comparison. For each tumour cohort, true positive driver elements were defined as elements called by at least three methods. The calculation of precision, recall and F1 score was then identical as for the CDS and splice site.

### Somatic copy number and SVs analysis

We used SCNA (including GISTIC2.0 results) and SV call sets released January 2017^70^. The copy number status (loss, neutral or gain) of a region is classified based on the difference between the absolute copy number of the region and the genome-wide ploidy of the donor. For gene-level SVs, we calculated the number of breakpoints per gene (including CDS, splice sites, UTRs and promoters) per donor.

### Differential expression analysis

We used the upper quartile normalised gene expression (FPKM-UQ) released May 2016^71^. When comparing the expression difference between two groups of donors, we fitted the following quasi-Poisson family GLM and then employed the likelihood ratio test to obtain *p* values for mutational status: FPKM-UQ ~ MUT + SCNA + [Tissue], where MUT is the mutational status (0 for unmutated donors and 1 for mutated donors), SCNA is the somatic copy number status (−1, 0 or 1 for copy number loss, neutral or gain, respectively) and Tissue is the tumour tissue type. The tissue type was only used for pan-cancer comparison for the adjustment of tumour types.

### WES data analysis

We obtained two WES data sets through the Genomic Data Common (GDC)^72^: TCGA-PAAD (35,321 somatic mutations across 180 samples) and TCGA-LIHA (56,208 somatic mutations across 364 samples). We chose public MuTect2 variants from GDC. Variant coordinates were lifted from hg38 to hg19 with the UCSC liftOver tool. Only CADD scores were used to detect drivers. For TCGA-PAAD, GBM models trained from Panc-AdenoCA of the PCAWG data were used. For TCGA-LIHA, GBM models trained from Liver-HCC were used.

### Reporting summary

Further information on research design is available in the Nature Research Reporting Summary linked to this article.

## Supplementary information

Peer Review File

Supplementary Information

Description of Additional Supplementary Files

Supplementary Data 1

Supplementary Data 2

Supplementary Data 3

Supplementary Data 4

Reporting Summary

## Data Availability

The source code for DriverPower (written mainly in Python 3) is available at GitHub [https://github.com/smshuai/DriverPower]. It is distributed under GNU General Public License 3.0, which allows for reuse and redistribution. Other software packages and bioinformatics tools used in this study are indicated in the corresponding method sections. The core computational pipelines used by the PCAWG Consortium for alignment, quality control and variant calling are available to the public at [https://dockstore.org/search?search=pcawg] under the GNU General Public License v3.0, which allows for reuse and distribution.

## Data availability

The data sets underpinning the analyses in the paper are detailed in Supplementary Table 1. Aligned sequencing data, as well as somatic and germline variant calls from PCAWG tumours, including SNVs, indels, copy number alterations and structural variants, are available for download at [https://dcc.icgc.org/releases/PCAWG]. Additional information on accessing the data, including raw read files, can be found at [https://docs.icgc.org/pcawg/data/]. In accordance with the data access policies of the ICGC and TCGA projects, most molecular, clinical and specimen data are in an open tier, which does not require access approval. To access potentially identification information, such as germline alleles and underlying sequencing data, researchers will need to apply to the TCGA Data Access Committee (DAC) via dbGaP [https://dbgap.ncbi.nlm.nih.gov/aa/wga.cgi?page=login] for access to the TCGA portion of the data set, and to the ICGC Data Access Compliance Office (DACO; [https://icgc.org/daco]) for the ICGC portion. In addition, to access somatic SNVs derived from TCGA donors, researchers will also need to obtain dbGaP authorisation.

In addition, the analyses in this paper used several data sets that were derived from the raw sequencing data and variant calls (Supplementary Table 1). The individual data sets are available at Synapse (https://www.synapse.org/), and are denoted with synXXXXX accession numbers (listed under Synapse ID); all these data sets are also mirrored at https://dcc.icgc.org, with full links, filenames, accession numbers and descriptions detailed in Supplementary Table 1. The data sets encompass: harmonised tumour histopathology annotations using a standardised hierarchical ontology (syn10389164); consensus somatic SNVs, MNVs and indels (syn7364923); gene expression profiles from RNA-sequencing data (syn5553991); genomic intervals used in driver region calls (syn5259890); driver region calls by each individual methods (syn7359546); consensus gene-level somatic copy number calls (syn8239175); three simulation data sets for somatic mutations (syn7187923, syn7436065 and syn7152699).
