# Supplementary material for: Combined burden and functional impact tests for cancer driver discovery using DriverPower
Source: Nat Commun. 2020 Feb 5;11:734. doi: 10.1038/s41467-019-13929-1 (PMC7002750; doi:10.1038/s41467-019-13929-1)
Supplement: Supplementary file 3 — Description of Additional Supplementary Files [file 41467_2019_13929_MOESM3_ESM.pdf]

Description of Additional Supplementary Files

File Name: Supplementary Data 1

Description: Summary of tumour cohorts. Names and sizes of tumour cohorts used in this study.

File Name: Supplementary Data 2

Description: Summary of genomic features. All feature names, URLs and feature importance scores are included in this table.

File Name: Supplementary Data 3

Description: Coding and Non-coding driver candidates. A complete list of coding and non-coding driver candidates detected by DriverPower from the PCAWG data.

File Name: Supplementary Data 4

Description: DriverPower-exclusive driver candidates. A complete list of DriverPower-exclusive driver candidates.
